# Solvent-free synthesis of novel *para*-menthane-3,8-diol ester derivatives from citronellal using a polymer-supported scandium triflate catalyst

**DOI:** 10.3762/bjoc.12.193

**Published:** 2016-09-19

**Authors:** Lubabalo Mafu, Ben Zeelie, Paul Watts

**Affiliations:** 1 Nelson Mandela Metropolitan University, University Way, Port Elizabeth, 6031, South Africa

**Keywords:** acylation, diesters, *para*-menthane-3,8-diol, PS-Sc(OTf)_3_

## Abstract

The use of natural resources as a chemical feedstock for the synthesis of added-value products is gaining interest; as such we report an environmentally friendly method for the synthesis of *para*-menthane-3,8-diol from natural citronellal oil in 96% yield, under solvent free aqueous conditions. The acylation of *para*-menthane-3,8-diol with various acid anhydrides over polymer-supported scandium triflate (PS-Sc(OTf)_3_) catalyst was subsequently developed, where both hydroxy groups of *para*-menthane-3,8-diol could be simultaneous acylated under mild reaction conditions to form the corresponding diesters in good yields. The advantages of this method include a simple procedure from natural resources, using solvent-free reaction conditions.

## Introduction

Although South Africa has a substantial petrochemical industry, the fine chemical industry is very small and most chemicals are imported. As such there is significant interest in the use of natural resources for the manufacture of added value products; ideally enabling the economy to become more self-sufficient by manufacturing advanced materials within the country. Furthermore, in the long term it is hoped that this will result in job creation and stimulate economic growth. The use of natural resources is gaining interest from a sustainability perspective, but clearly it is necessary to develop protocols that are as environmentally friendly and sustainable as possible.

**Scheme 1:**
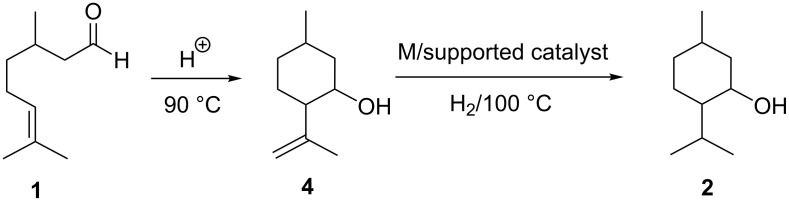
Synthesis of menthol.

The terpene, citronellal (3,7-dimethyl-6-octenal, **1**) is widely used as a feedstock material in the synthesis of fine chemicals such as menthol (**2**) and *para*-menthane-3,8-diol (**3**) [1–2]. These chemical derivatives have a wide range of uses in pharmaceuticals, cosmetics, toothpastes, insect repellents, cleaning agents and other products [3]. The synthesis of menthol involves the acid-catalysed cyclisation of **1** to form isopulegol **4** as a stable intermediate. The latter is further hydrogenated over a metal-supported catalyst to yield the *cis* and *trans* isomers of menthol **2** (Scheme 1) [4].

**Scheme 2:**
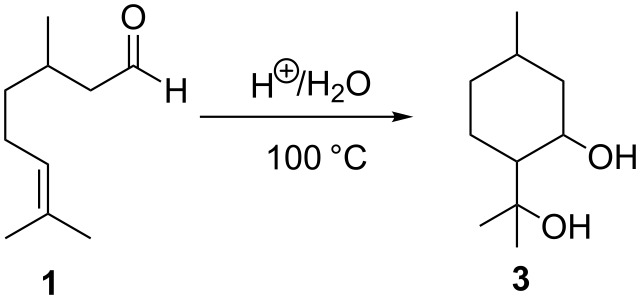
Synthesis of *para*-menthane-3,8-diol.

Alternatively the acid-catalysed hydration of citronellal (**1**) results in the synthesis of the *cis* and *trans* isomers of *para*-menthane-3,8-diol (**3**, Scheme 2). This chemical derivative is well-known as an active insect repellent and can be found naturally as a minor component of citriodora oil [2]. Considering the chemical structure of **3**, two reactive hydroxy groups are present which can undergo an organic transformation, such as acylation, to yield natural bio-based compounds.

The acylation of alcohols, thiols and amines is a fundamental reaction in organic synthesis. It is mostly used to protect these functional groups in multi-step synthesis processes. The acylation reaction is typically carried-out with activated carboxylic acid derivatives such as acid anhydrides [5], acyl halides [6], acyl imidazoles or acyl ureas [7]. Acylation of alcohols in particular, provides a cheap and effective method for the synthesis of esters with potential applications in pharmaceutical products such as fragrances, flavours, surfactants or solvents [8–9]. Generally, these reactions are done in the presence of amines such as pyridine, triethyl amine or 4-(dimethylamino)pyridine [7] homogeneous Lewis acid catalysts (AlCl_3_, BF_3_, TaCl_5_) [10] or inorganic acids are also used [11]. Recent publications have reported scandium triflate (Sc(OTf)_3_) to be an effective catalyst in the acylation of alcohols with acid anhydrides and the reaction can be carried out under mild conditions [10–11].

As part of our research investigations, we report the synthesis of novel diester derivatives of *para*-menthane-3,8-diol (PMD, **3**). These diester derivatives are currently being studied within our group for a variety of applications. The synthesis method involves the acylation of **3** with various acid anhydrides. The synthesis method also employs a polymer-supported scandium triflate as a water resistant and environmentally friendly acid catalyst.

## Results and Discussion

### Synthesis *para*-menthane-3,8-diol from citronellal

*para*-Menthane-3,8-diol (**3**) was synthesised according to our earlier developed procedure, which has not been reported in open literature. The synthesis procedure involves the acid-catalysed cyclisation of **1** in aqueous sulfuric acid (Scheme 2) at 100 °C. After which the oil simply separates from the aqueous acid to furnish the product. After recrystallization, the final product **3** was obtained as white crystals in 96% isolated yield.

#### Synthesis of diester derivatives

**Scheme 3:**
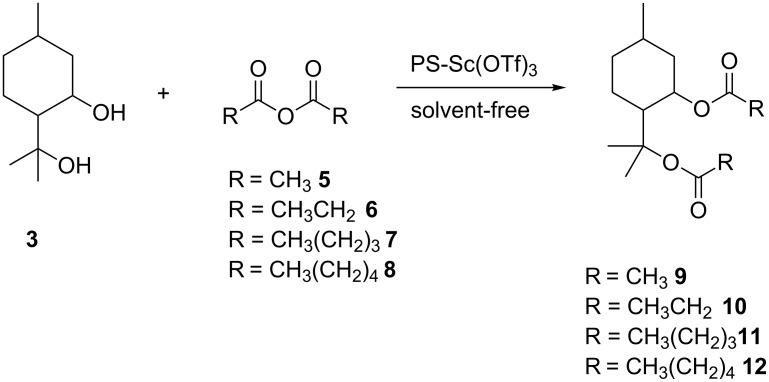
Synthesis of *para*-menthane diester derivatives.

Having successfully demonstrated the synthesis of **3**, the investigation was extended to the preparation of diester derivatives of **3** via the acylation reaction with acid anhydrides (Scheme 3). It needs to be clarified that earlier attempts to perform classic esterifications by reaction of the alcohols with a carboxylic acid were not particularly successful, as very complex reaction mixtures were produced as a result of dehydration of the starting material. During the study, various acid anhydrides such as acetic **5**, propionic **6**, pentanoic **7** and hexanoic anhydride **8** were used to prepare the corresponding diester derivatives **9–12**.

To afford an environmentally-friendly process, a polymer-bound scandium triflate (PS-Sc(OTf)_3_) catalyst was used. Moreover, all the reactions were carried-out under solvent-free conditions. Reaction parameters such as temperature, reaction time and reagent molar ratio were studied towards the substrate conversion and product selectivity.

#### Effect of reaction temperature and reaction time

**Figure 1:**
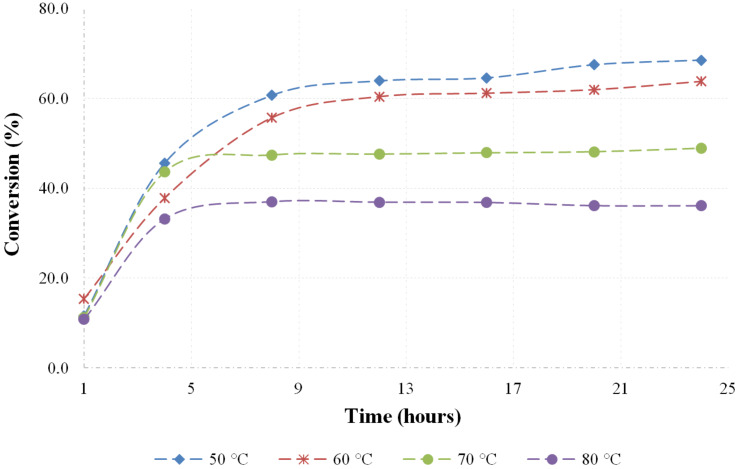
PMD conversion using stoichiometric quantities of acetic anhydride.

In order to determine the effect of temperature and reaction time towards the diester formation, the acylation reaction of *para*-menthane-3,8-diol (**3**) with acetic anhydride **5** was carried out using equimolar amounts of reagent (i.e. 2 equivalents of anhydride per mole of diol). The reaction was conducted at various temperatures ranging from 50 to 80 °C, while other parameters such as stoichiometric ratio, reaction time, catalyst loading and stirring rate were kept constant. The reactions were followed by taking samples at hourly time intervals and quantified by gas chromatography. Figure 1 shows the graphical presentation of PMD **3** conversion to the desired product at various temperatures.

It can be seen on the graph in Figure 1 that the PMD **3** conversion to diesters has its optimum at lower temperatures. When the reaction is operated at 70 °C and above, dehydration of the substrate starts to occur, leading to complex reaction mixtures.

**Figure 2:**
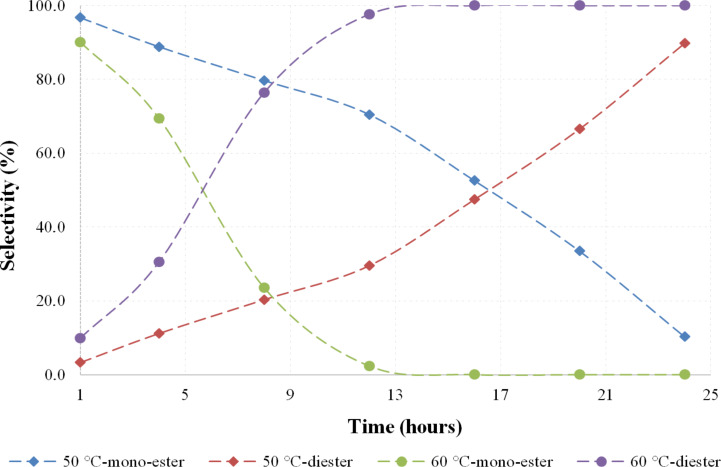
Product distribution as a function of time.

When considering the diester selectivity (Figure 2), a rapid acetylation of the secondary hydroxy group is evident at short reaction times and lower reaction temperatures between 50 and 60 °C [12].

**Figure 3:**
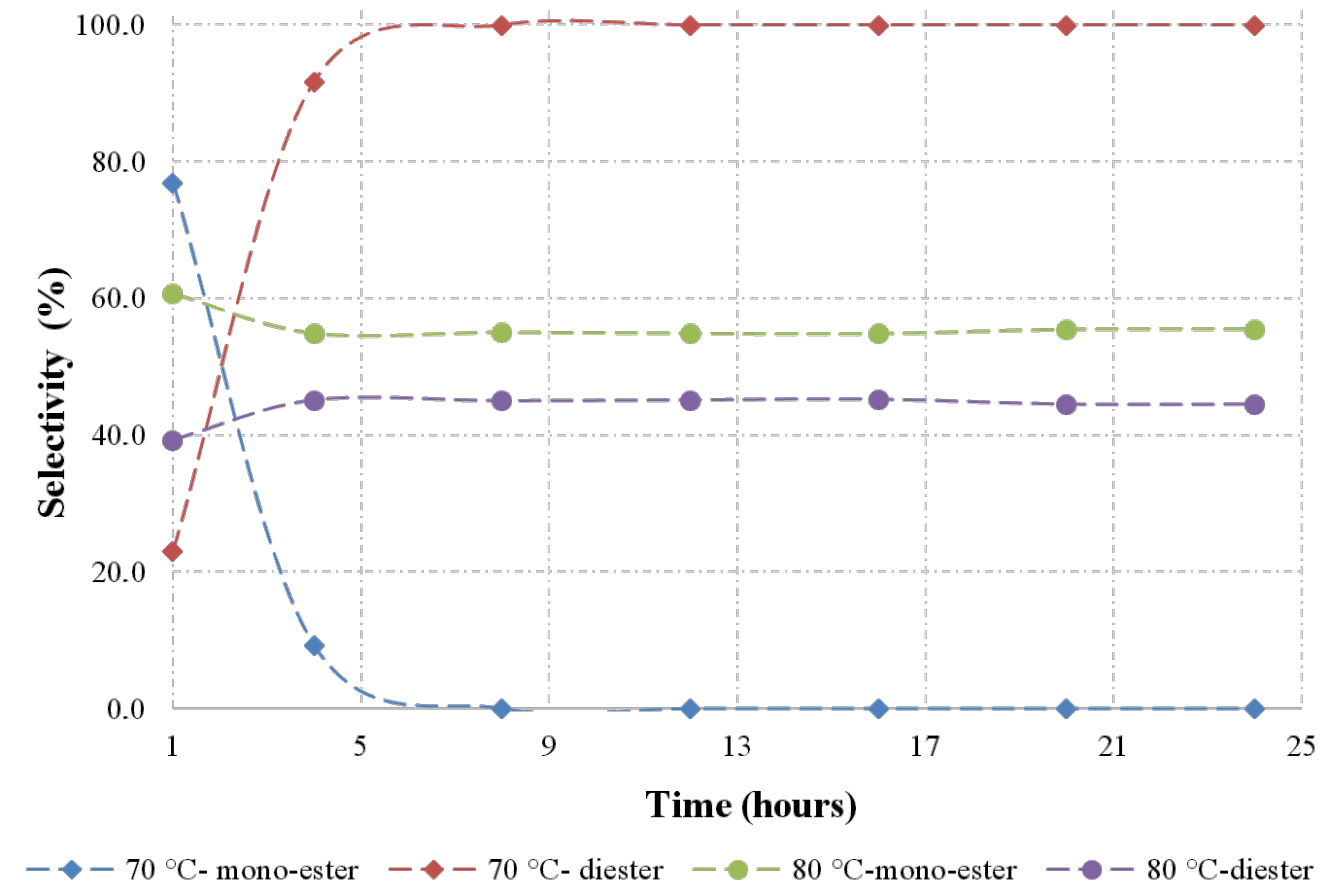
Product distribution as a function of time.

Further increase in the reaction time leads to the acetylation of the less reactive tertiary hydroxy group. Consequently, the diacetate **9** becomes the major product of the reaction. As demonstrated on the graph, the slow reaction rate is evident at lower temperature [12]. A slight increase in temperature improves the reaction rate, as well as the selectivity to diacetate **9**. On further increase in temperature to 70 °C (Figure 3), the decomposition of the starting material is observed and these conditions are clearly unfeasible. However, diacetate selectivity is achieved in shorter residence time. Above 80 °C, poor conversion of **3** and selectivity of **9** are evident. This clearly indicates that this reaction does not tolerate reaction temperatures higher as 70 °C.

The optimum conditions for the reaction are found to be a temperature of 60 °C and a reaction time of 12 hours. At these conditions, the reaction gives a substrate conversion of 60% and 100% diacetate selectivity, without any dehydration or mono acetate **13** formation.

#### Effect of molar ratio

**Figure 4:**
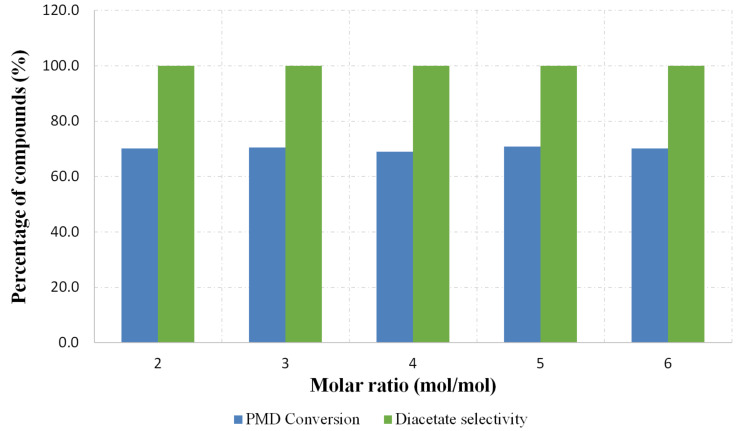
Effect of molar ratio in product distribution.

The effects of the molar ratio toward the conversion of **3** and the selectivity for **9** were further evaluated at the optimum conditions as highlighted above. The acetic anhydride to *para*-menthane-3,8-diol molar ratio ranging from 2 to 6 were used for the study and other reaction parameters were kept constant. Figure 4 shows the PMD **3** conversion and the selectivity for the formation of **9**, where the graph clearly shows that the use of excess anhydride **5** does not enhance the substrate conversion.

It is clearly shown that when performing an acetylation reaction with 2:1 molar ratio, the reaction affords the same results as when excess anhydride **5** is used. Moreover, the minimum use of the acetylating reagent helps to reduce the formation of carboxylic acid as the by-product. As a consequence, a more cost-effective and environmentally-friendly process is achieved. These results are found to be in agreement to those of Gagea et al., when they demonstrated the effect of molar ratio of acid anhydride-to-alcohol over the silica embedded-triflate catalysts [8]. All the experiments were conducted using the same catalyst and we observed no degradation in performance. However, we are conducting further studies to see how long the reaction could be conducted from a production perspective.

#### Synthesis of propyl, pentyl and hexyl diester derivatives

Having successfully synthesised the diacetate **9**, other acid anhydrides were evaluated in the process to yield diester derivatives. These include the propionic, pentanoic and hexanoic anhydrides, respectively. The following optimum conditions were used for the study; temperature of 60 °C, reaction time of 12 hours, catalyst loading of 0.3 g and molar ratio of 1:2. Table 1 below shows the acylation results obtained with various acid anhydrides.

**Table 1:** Synthesis of propyl, pentyl and hexyl derivatives.

Reagent	PMD conv. (%)	Monoester sel. (%)	Diester sel. (%)

propionic anhydride	70.5	0	65.8
pentanoic anhydride	69.6	10	63.2
hexanoic anhydride	70.1	16	60.4

**Scheme 4:**
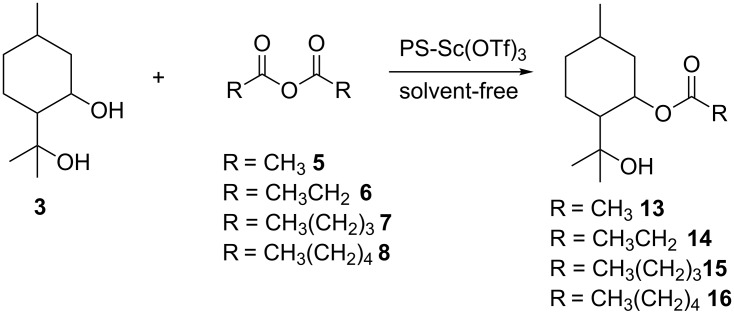
Synthesis of *para*-menthane mono-ester derivatives.

It can be seen in Table 1, that the substrate conversion has remained unchanged under these conditions, with the remaining PMD being unreacted. On the other hand, the acid anhydrides with shorter carbon chain appear to be more reactive to yield the diester derivatives, with more monoester being formed as the length of the chain increased. In the case of propionic anhydride, its mono-ester derivative **14** is completely converted into its corresponding diester derivative **10** in 12 hours of reaction time (Scheme 4).

However, the change in the carbon chain length of acid anhydride to C_5_ or C_6_, leads to a significant decrease in the reactivity towards the tertiary hydroxy group. As a result, the monopentanoate **15** and monohexanoate **16** are found to be present in about 10% yield when the same procedure was used.

## Conclusion

In conclusion, we have successfully demonstrated the synthesis of novel diester derivatives of *para*-menthane-3,8-diol. The process involves the use of a solvent-free system and the reaction occurs at mild conditions. In addition, the use of polymer-bound scandium triflate has been shown to be very efficient in the acylation reaction. Moreover, the catalyst was reusable in the process without significant change towards the substrate conversion and product selectivity. Using the methodology described herein, further studies are currently underway within our laboratory to optimise the developed method in a continuous flow process.

## Experimental

### Materials and methods

All the reagents (analytical grade) were purchased from Sigma-Aldrich and were used without purification. The citronellal feedstock material was purchased from Germany (Chemical point). The quantification of product mixtures were performed on an Agilent Gas chromatograph, equipped with a flame ionization detector, Econocap-5 column (film thickness 0.25 µm; internal diameter 0.25 mm; length 30 m) and ultra-high purity nitrogen (99.999%) carrier gas. The samples were analysed by using the following method; injector temperature 270 °C, nitrogen flow rate 0.5 mL·min^−1^, oven temperature 70 °C for 5 min and then ramped to 270 °C at 10 °C min^−1^ an final hold-up time of 5 min. All NMR spectra were recorded as solutions in deuterochloroform (CDCl_3_) using tetramethylsilane (TMS) as an internal standard. The spectra were recorded on a Bruker Ultrashield Plus spectrometer, which was operated at 400 MHz for proton and 100 MHz for carbon. The chemical shift values for all spectra are given in parts per million (ppm) with coupling constants in Hertz (Hz). The following observations are used to report NMR data; s = singlet, d = doublet, t = triplet, br s = broad singlet, m = multiplet and C_0_ = quaternary carbon. Gas chromatography (GC–MS) spectrometry was performed on a HP F5890 series LL plus gas chromatograph coupled to an HP 5972 series mass selective detector. The GC was equipped with a HP-5 MS capillary column (30 mm × 0.25 mm i.d.) and ultra-high purity helium (99.999%) carrier gas. The samples were analysed by using the following method; injector temperature 250 °C, helium flow rate 0.1 mL·min^−1^, oven temperature 70 °C for 5 min and then ramped to 280 °C at 10 °C min^−1^ with split flow ratio of 60 mL·min^−1^. The FTIR characteristic peaks were recorded on a Bruker Platinum Tensor 27 spectrophotometer with an ATR fitting. The analyses of samples were recorded in the range 4000–600 cm^−1^ and the peaks are reported in wavenumbers (cm^−1^). The solid and liquid samples were analysed without any modification. The boiling points of the compounds were measured using a simulated distillation (Agilent SimDis FAST2887) instrument fitted with a CAP. EXT. 2887/AC column (film thickness 0.88 µm; internal diameter 0.53 mm; length 10 m).

### Experimental procedures

#### Synthesis of *para*-menthane-3,8-diol from citronellal

Citronellal (30.08 g, 0.193 mol) was added into stirred dilute sulphuric acid (140 g, 0.0076 mol of a 0.3% (v/v)) solution at a temperature of 100 °C. After 4 hour of stirring the aqueous phase was separated from the organic oil phase. The organic phase was neutralised with 50 mL of 2.5% (v/v) sodium hydrogen carbonate (NaHCO_3_) solution to remove the remains of sulphuric acid catalyst and dried (MgSO_4_). The product was recrystallized from *n*-hexane at −18 °C for 24 hours. *p*-Menthane-3,8-diol (**3**) was obtained as white crystals (96%). ^1^H NMR (400 MHz, CDCl_3_, ppm) δ 0.80–0.96 (m, 3H), 0.89–0.90 (m, 1H), 0.99–1.03 (m, 2H), 1.11–1.19 (m, 3H), 1.33 (s, 3H), 1.66–1.68 (m, 2H), 1.72–1.82 (m, 3H), 3.40 (s, 2H) and 4.38 (br s, 1H); ^13^C NMR (100 MHz, CDCl_3_, ppm) δ 20.2, 22.1, 25.5, 28.6, 29.9, 34.8, 42.4, 49.2, 67.7 and 73.2; FTIR (cm^−1^): 3220, 2941, 2911, 1158 and 931; *m/z* (CI) 172 (M^+^, 1), 157 (9), 139 (20), 96 (50), 81 (100) and 59 (90); GC *t*_R_ = 15.0 min.

#### General procedure for the synthesis of diester derivatives

*para*-Methane-3,8-diol (**3**, 5.0 g, 0.029 mol) and an appropriate molar equivalent of acid anhydride were transferred into the reactor concurrently. Both reagents were stirred and heated at 60 °C for 10 minutes. The homogeneous mixture was achieved and 0.3 g of polymer-bound scandium triflate (PS-Sc(OTf)_3_) catalyst was added into the reaction mixture. The reaction was heated at the appropriate temperature for 24 hours, while followed by sampling at an hourly interval. Upon the completion of the reaction, the catalyst was separated from the product mixture by filtration and the acid byproduct was removed by vacuum distillation. The obtained crude sample was subsequently purified by column chromatography hexane/EtOAc (98:2).

**Diacetate 9:** The reaction was carried out in accordance with the general procedure using *para*-menthane-3,8-diol (**3**, 5.0 g, 0.029 mol) and acetic anhydride (**5**, 7.4 g, 0.073 mol) to give the title compound **9** as viscous colourless oily liquid, bp 288 °C, (6.8 g, 91%); ^1^H NMR (400 MHz, CDCl_3_, ppm) δ 0.78–0.79 (m, 3H), 0.89–1.04 (m, 2H), 1.35 (br d, *J* = 12 Hz, 6H), 1.53–1.60 (m, 3H), 1.72 (d, *J* = 16 Hz, 1H), 1.82 (d, *J* = 12 Hz, 1H), 1.88 (s, 3H), 1.96 (s, 3H), 2.02–2.08 (m, 1H) and 5.17 (br s, 1H); ^13^C NMR (100 MHz, CDCl_3_, ppm) δ 21.4, 21.9, 22.2, 22.3, 24.0, 25.1, 26.6, 34.6, 39.4, 47.3, 69.8, 84.2, 169.9 and 170.3; FTIR (cm^−1^): 2949, 1728, 1180 and 1144; *m/z* (CI) 256 (M^+^, 1), 197 (78), 137 (71), 95 (62) and 81 (100); GC *t*_R_ = 17.8 min.

**Dipropionate 10:** The reaction was carried out in accordance with the general procedure using *para*-menthane-3,8-diol (**3**, 5.0 g, 0.029 mol) and propionic anhydride (**6**, 9.4 g, 0.073 mol) to give the title compound **10** as viscous colourless oily liquid, bp 319 °C, (8.04 g, 97%); ^1^H NMR (400 MHz, CDCl_3_, ppm) δ 0.78–0.86 (m, 3H), 0.89–0.92 (m, 1H), 0.95–0.99 (m, 4H), 1.01–1.07 (m, 3H), 1.34 (br d, *J* = 12 Hz, 6H), 1.50–1.60 (m, 3H), 1.72 (br d, *J* =12 Hz, 1H), 1.83 (d, *J* = 12 Hz, 1H), 2.04 (d, *J* = 12 Hz, 1H), 2.03–2.27(m, 4H), and 5.18 (br s, 1H); ^13^C NMR (100 MHz, CDCl3, ppm) δ 9.2, 22.0, 22.1, 24.1, 25.2, 26.7, 28.1, 28.8, 34.7, 39.5, 47.6, 50.0, 69.7, 84.1, 173.4 and 173.9; FTIR (cm^−1^): 2946, 1728, 1169 and 1143; *m/z* (CI) 284 (M^+^, 1), 211.4 (10), 136 (22), 81 (23) and 57 (100); GC *t*_R_ = 19.8 min.

**Dipentanoate 11:** The reaction was carried out in accordance with the general procedure using *para*-menthane-3,8-diol (**3**, 5.0 g, 0.029 mol) and pentanoic anhydride (**7,** 13.5 g, 0.073 mol) to give the title compound **11** as viscous colourless oily liquid, bp 363 °C, (10.2 g, 95%); ^1^H NMR (400 MHz, CDCl_3_, ppm) δ 0.78–0.84 (m, 9H), 0.92–1.04 (m, 1H), 1.25–1.28 (m, 5H), 1.32 (br d, *J* = 16 Hz, 6H), 1.48–1.56 (m, 7H), 1.72 (br d, *J* = 12 Hz, 1H), 1.84 (d, *J* = 16 Hz, 1H), 2.04 (d, *J* = 8 Hz, 1H), 2.12–2.22 (m, 4H), and 5.18 (br s, 1H); ^13^C NMR (100 MHz, CDCl_3_, ppm) δ 13.6, 13.7, 14.05, 22.2, 22.2, 22.3, 24.1, 24.6, 24.6, 25.1, 26.7, 27.0, 27.1, 47.4, 47.6, 69.7, 84.1, 84.3, 172.9 and 173.2; FTIR (cm^−1^): 2954, 1727, 1169 and 1143; *m/z* (CI) 341 (M^+^, 8), 281 (27), 207 (30), 93 (18), 85 (47) and 73 (100); GC *t*_R_ = 22.5 min.

**Dihexanoate 12:** The reaction was carried out in accordance with the general procedure using *para*-menthane-3,8-diol (**3**, 5.0 g, 0.029 mol) and hexanoic anhydride (**8**, 15.6 g, 0.073 mol) to give the title compound **12** as viscous colourless oily liquid, bp 395 °C, (10.4 g, 97%); ^1^H NMR (400 MHz, CDCl3, ppm) δ 0.76–0.85 (m, 10H), 0.94–1.98 (m, 2H), 1.08–1.26 (m, 6H), 1.38 (d, *J* = 4 Hz, 1H), 1.54–157 (m, 5H), 1.62–1.77 (m, 6H), 1.86–1.94 (m, 3H), 2.19–2.23 (m, 3H), 4.64–4.73 (m, 2H) and 5.24 (br s, 1H); ^13^C NMR (100 MHz, CDCl_3_, ppm) δ 13.8, 13.8, 21.9, 22.2, 22.3, 24.1, 24.6, 24.6, 25.1, 26.7, 31.2, 31.3, 34.7, 34.8, 35.5, 39.5, 47.5, 69.6, 77.4, 84.0, 172.6 and 173.1; FTIR (cm^−1^): 2952, 1728, 1128 and 1107; *m/z* (C.I) 369 (M^+^, 1), 253 (27), 136 (34) and 99 (100); GC *t*_R_ = 26.4 min.

#### General procedure for the synthesis of monoester derivatives

*para*-Methane-3,8-diol (**3**, 5.0 g, 0.029 mol) and an appropriate molar equivalence of acid anhydride were transferred into the reactor concurrently. Both reagents were stirred and heated at 60 °C for 10 minutes. The homogeneous mixture was achieved and 0.3 g of polymer-bound scandium triflate (PS-Sc(OTf)_3_) catalyst was added into the reaction mixture. The reaction was stirred 60 °C for 24 hours, while followed by sampling at an hourly interval. Upon the completion of the reaction, the catalyst was separated from the product mixture by filtration and the acid was removed by distillation. The obtained crude sample was purified by column chromatography hexane/EtOAc (98:2). The colourless oily products were analysed.

**Monoacetate 13:** The reaction was carried out in accordance with the general procedure using *para*-menthane-3,8-diol (**3**, 5.0 g, 0.029 mol) and acetic anhydride (**5**, 4.4 g, 0.044 mol) to give the title compound **13** as viscous colourless oily liquid, bp 275 °C, (5.3 g, 85%); ^1^H NMR (400 MHz, CDCl_3_, ppm) δ 0.79–0.99 (m, 5H), 1.09 (br d, *J* = 12 Hz, 6H), 1.32 (d, *J* = 12 Hz, 1H), 1.54–1.67 (m, 3H), 1.74 (br d, *J* = 4 Hz, 1H), 1.88 (d, *J* = 16 Hz, 1H), 1.98 (br s, 3H), 2.29 (br s, 1H) and 5.29 (br s, 1H); ^13^C NMR (100 MHz, CDCl_3_, ppm) δ 21.5 21.9, 22.0, 26.5, 27.5, 28.5, 34.7, 39,4, 50.0, 71.1, 71.8 and 170.5; FTIR (cm^−1^): 3435, 2948, 1734, 1455, 1241 and 1080; *m/z* (CI) 214 (M^+^, 1), 197 (100), 137 (70), 95 (65), 81 (100) and 59 (48); GC *t*_R_ = 16.2 min.

**Monopropionate 14:** The reaction was carried out in accordance with the general procedure using *para*-menthane-3,8-diol (**3**, 5.0 g, 0.029 mol) and propionic anhydride (**6**, 5.7 g, 0.044 mol) to give the title compound **14** as viscous colourless oily liquid, bp 282 °C, (5.8 g, 87.6%); ^1^H NMR (400 MHz, CDCl_3_, ppm) δ 0.79 (d, *J* = 8 Hz, 3H), 0.6–1.11 (m, 10H), 1.33 (d, *J* = 16 Hz, 1H), 1.54–1.64 (m, 3H), 1.74 (d, *J* = 12 Hz, 1H), 1.87 (d, *J* = 12 Hz, 1H), 2.23–2.29 (m, 3H), 3.77 (br s, 1H) and 5.30 (br s, 1H); ^13^C NMR (100 MHz, CDCl_3_, ppm) δ 9.0, 21.9, 22.1, 26.6, 27.5, 28.2, 28.5, 34.7, 39.5 49.9, 71.0, 72.1, and 173.9; FTIR (cm^−1^): 3425, 2947, 2870, 2847, 1730, 1375, 1279 and 1191; *m*/*z* (CI) 228 (M^+^, 1), 211 (10), 136 (20), 81 (25) and 57 (100); GC *t*_R_ = 17.3 min.

**Monopentanoate 15:** The reaction was carried out in accordance with the general procedure using *para*-menthane-3,8-diol (**3**, 5.0 g, 0.029 mol) and pentanoic anhydride (**7**, 8.1 g, 0.044 mol) to give the titled compound **15** as viscous colourless oily liquid, bp 290 °C, (6.7 g, 90.1%); ^1^H NMR (400 MHz, CDCl_3_, ppm); δ 0.78–0.85 (m, 7H), 1.00 (d, *J* = 12 Hz, 6H), 1.04–1.11 (m, 3H), 1.12–1.33 (m, 5H), 1.68 (d, *J* = 12 Hz, 1H), 1.74 (d, *J* = 12 Hz, 1H), 1.86–2.08 (m, 2H), 2.09–2.23 (m, 2H) and 5.29 (br s, 1H); ^13^C NMR (100 MHz, CDCl_3_, ppm) δ 13.6, 22.0, 22.1, 22.2, 22.6, 26.9, 27.6, 28.6, 34.6, 34.7, 39.5, 49.8, 71.0, 71.9 and 173.3; FTIR (cm^−1^): 3436, 2954, 2929, 2870, 1730, 1181, 1145 and 996; *m/z* (CI) 256 (M^+^, 1), 136 (60), 86 (100), 57 (80), 29 (10); GC *t*_R_ = 18.1 min.

**Monohexanoate 16** The reaction was carried out in accordance with the general procedure using *para*-menthane-3,8-diol (**3**, 5.0 g, 0.029 mol) and hexanoic anhydride (**8**, 9.3 g, 0.044 mol) to give the titled compound **16** as viscous colourless oily liquid, bp 297 °C, (6.9 g, 88.1%); ^1^H NMR (400 MHz, CDCl3, ppm) δ 0.82–1.05 (m, 9H), 1.13 (br d, *J* = 16 Hz, 6H), 1.28–1.37 (m, 3H), 1.55–1.71 (m, 5H), 1.78 (br d, *J* = 16 Hz, 1H), 1.91 (d, *J* = 16 Hz, 1H), 2.26–2.31 (m, 4H) and 5.32 (br s, 1H); ^13^C NMR (100 MHz, CDCl3, ppm) δ 13.6, 21.9, 22.1, 22.2, 26.6, 27.6, 28.6, 34.6, 34.7, 39.5, 49.9, 70.9, 71.9 and 173.3; FTIR (cm^−1^): 3436, 2952, 2931, 2870, 1729, 1181 and 1146; *m/z* (CI) 270 (M^+^, 1), 253 (10), 136 (20) and 99 (100); GC *t*_R_ = 19.2 min.

## Supporting Information

File 1 NMR, IR and GC–MS spectra of synthesized compounds.

## References

[1] da Silva K A, Robles-Dutenhefner P A, Sousa E M B, Kozhevnikova E F, Kozhevnikov I V, Gusevskaya E V (2004). Catal Commun.

[2] Drapeau J, Rossano M, Touraud D, Obermayr U, Geier M, Rose A, Kunz W (2011). C R Chim.

[3] Imachi S, Owada K, Onaka M (2007). J Mol Catal A: Chem.

[4] Nie Y, Niah W, Jaenicke S, Chuah G-K (2007). J Catal.

[5] Heravi M M, Behbahani F K, Bamoharram F F (2007). ARKIVOC.

[6] Sharma R K, Gulati S (2012). J Mol Catal A: Chem.

[7] Chandra K L, Saravanan P, Singh R K, Singh V K (2002). Tetrahedron.

[8] Pârvulescu A N, Gagea B C, Poncelet G, Pârvulescu V I (2006). Appl Catal, A.

[9] Tashiro D, Kawasaki Y, Sakaguchi S, Ishii Y (1997). J Org Chem.

[10] Dumeunier R, Markó I E (2004). Tetrahedron Lett.

[11] Mpuhlu B (2007). Synthesis of p-menthane-3,8-diol.

[12] Firouzabadi H, Iranpoor N, Farahi S (2008). J Mol Catal A: Chem.

