# Supplementary material for: Solvent-free synthesis of novel para-menthane-3,8-diol ester derivatives from citronellal using a polymer-supported scandium triflate catalyst
Source: Beilstein J Org Chem. 2016 Sep 19;12:2046–54. doi: 10.3762/bjoc.12.193 (PMC5082460; doi:10.3762/bjoc.12.193)
Supplement: File 1 — NMR, IR and GC–MS spectra of synthesized compounds. [file Beilstein_J_Org_Chem-12-2046-s001.pdf]

Supporting Information

for

Solvent-free synthesis of novel *para*-menthane-3,8-diol ester derivatives from citronellal using a polymer-supported scandium triflate catalyst

Lubabalo Mafu, Ben Zeelie and Paul Watts*

Address: Nelson Mandela Metropolitan University, University Way, Port Elizabeth, 6031, South Africa

Email: Paul Watts - Paul.Watts@nmmu.ac.za

*Corresponding author

1. STARTING MATERIAL AND OIL COMPONENTS S3
1.1. CITRONELLAL S3
FTIR- spectrum....... S3
GC–MS spectrum S3
^{1}H NMR spectrum S4
^{13}C NMR spectrum S5
1.2. ISOPULEGOL S6
FTIR spectrum....... S6
GC–MS spectrum S6
^{1}H NMR spectrum S7
^{13}C NMR spectrum S8
1.3. PARA-MENTHANE-3,8-DIOL S9
FTIR spectrum....... S9
GC–MS spectrum S9
^{1}H NMR spectrum S10
^{13}C NMR spectrum S11
2. PARA-MENTHANE-3,8-DIESTER DERIVATIVES S12
2.1. MONO-ACETATE....... S12
FTIR spectrum....... S12
GC–MS spectrum S12
^{1}H NMR spectrum S13
^{13}C NMR spectrum S14
2.2. DI-ACETATE S15
FTIR spectrum....... S15
GC–MS spectrum S15
^{1}H NMR spectrum S16

^{13}C NMR spectrum S17
2.3. MONO-PROPIONATE S18
FTIR spectrum S18
GC–MS spectrum S18
^{1}H NMR spectrum S19
^{13}C NMR spectrum S20
2.4. DI-PROPIONATE S21
FTIR spectrum S21
GC–MS spectrum S21
^{1}H NMR spectrum S22
^{13}C NMR spectrum S23
2.5. MONO-PENTANOATE S24
FTIR spectrum S24
GC–MS spectrum S24
^{1}H NMR spectrum S25
^{13}C NMR spectrum S26
2.6. DI-PENTANOATE S27
FTIR spectrum S27
GC–MS spectrum S27
^{1}H NMR spectrum S28
^{13}C NMR spectrum S29
2.7. MONO-HEXANOATE S30
FTIR spectrum S30
GC–MS spectrum S30
^{1}H NMR spectrum S31
^{13}C NMR spectrum S32
DI-HEXANOATE S33
FTIR spectrum S33
GC–MS spectrum S33
^{1}H NMR spectrum S34
^{13}C NMR spectrum S35

1. Starting material and oil components

1.1. Citronellal

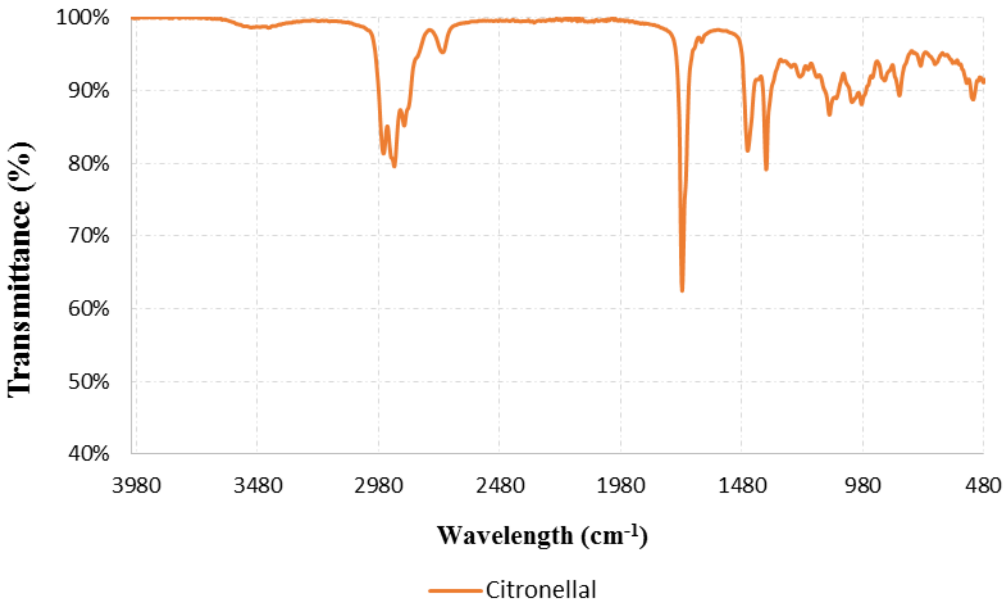

FTIR- spectrum

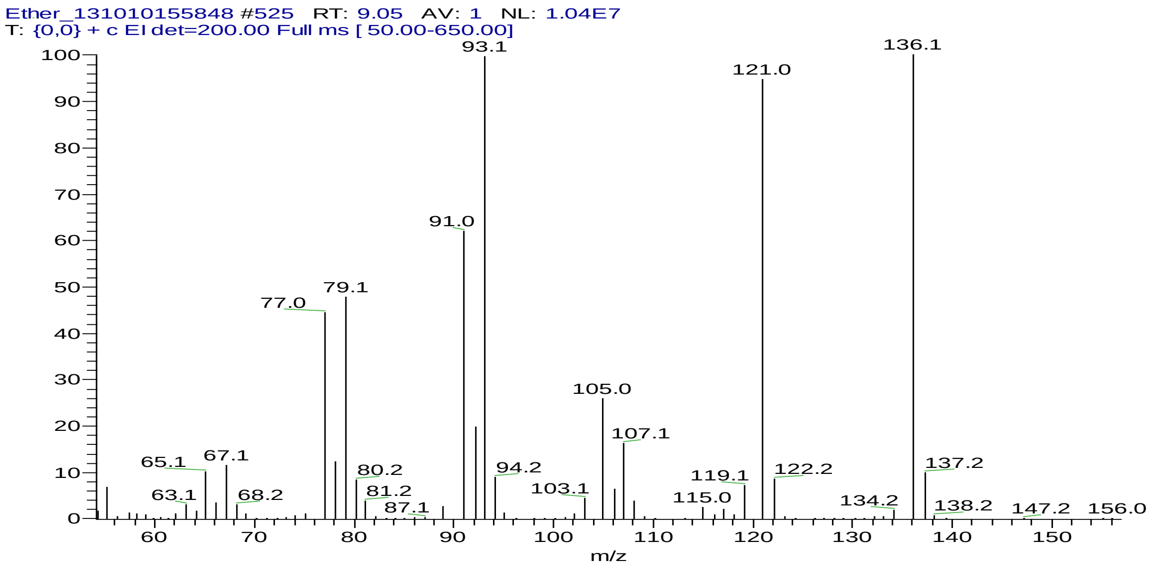

GC–MS spectrum

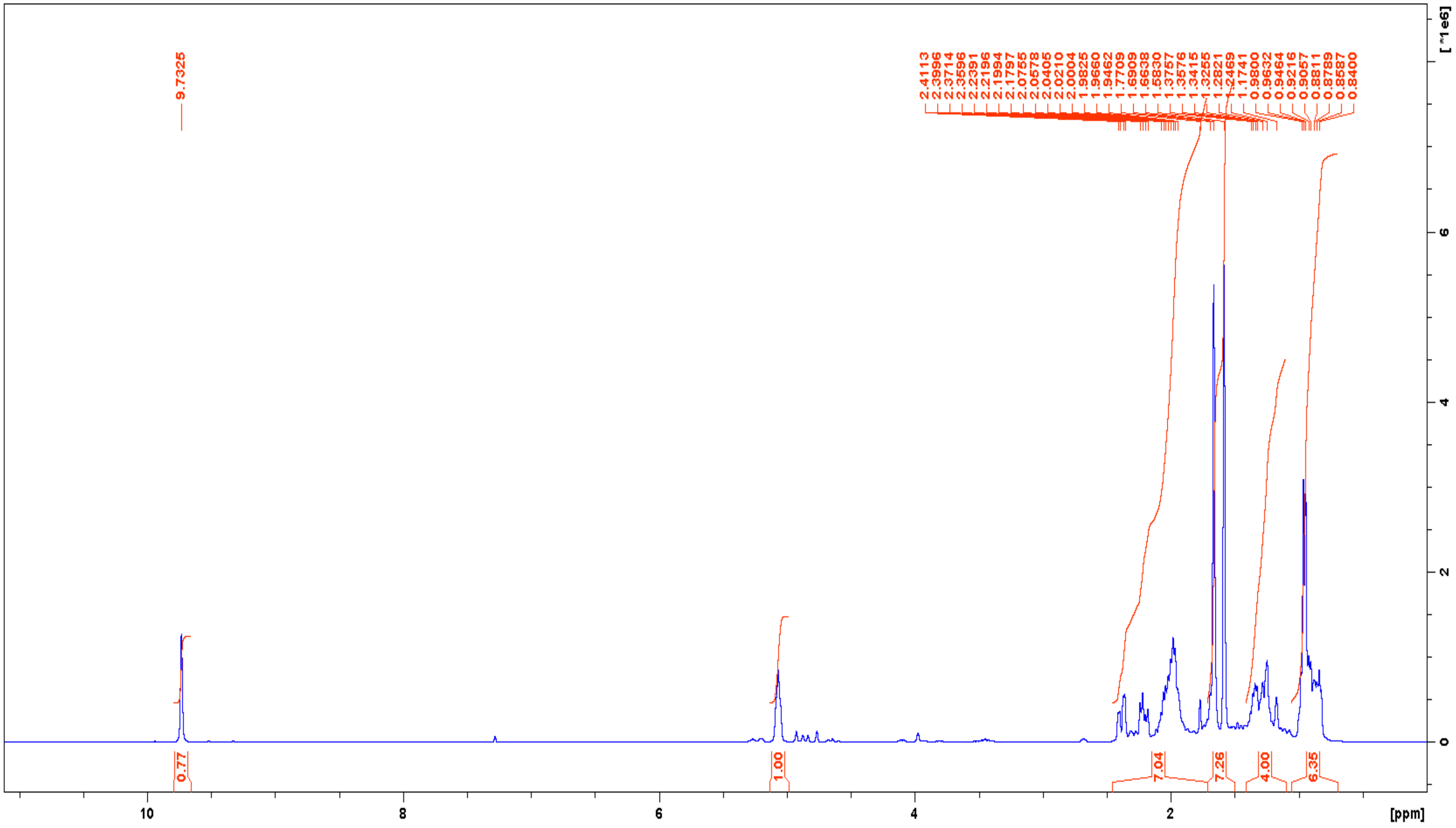

^{1}H NMR spectrum

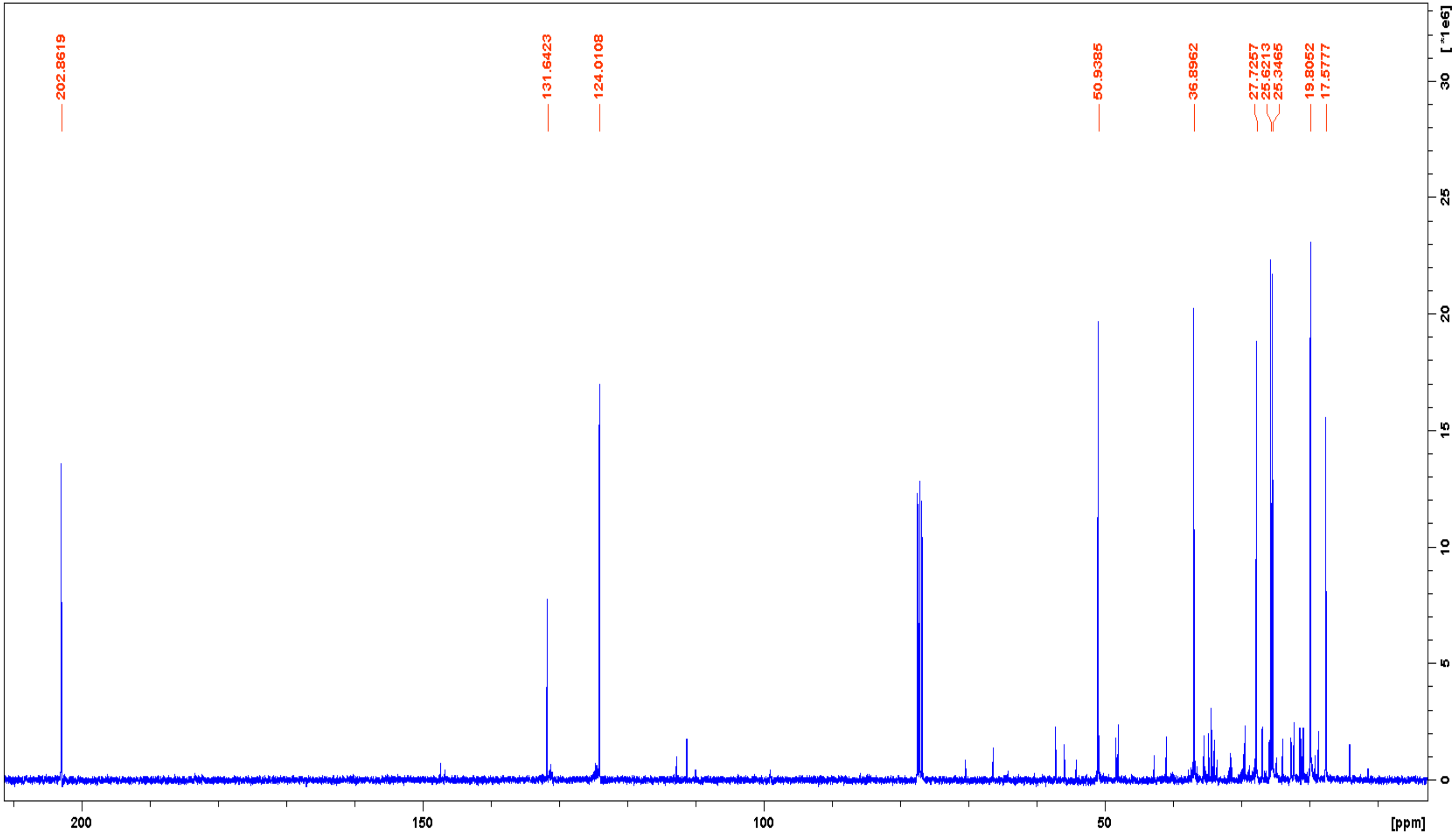

^{13}**C NMR spectrum**

1.2. Isopulegol

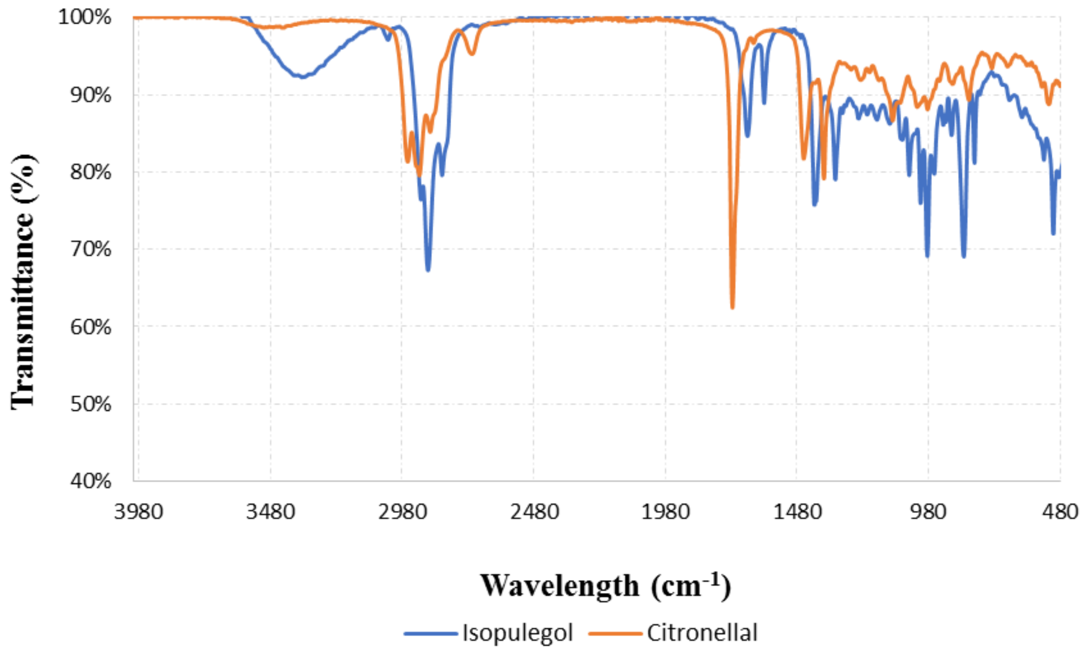

FTIR spectrum

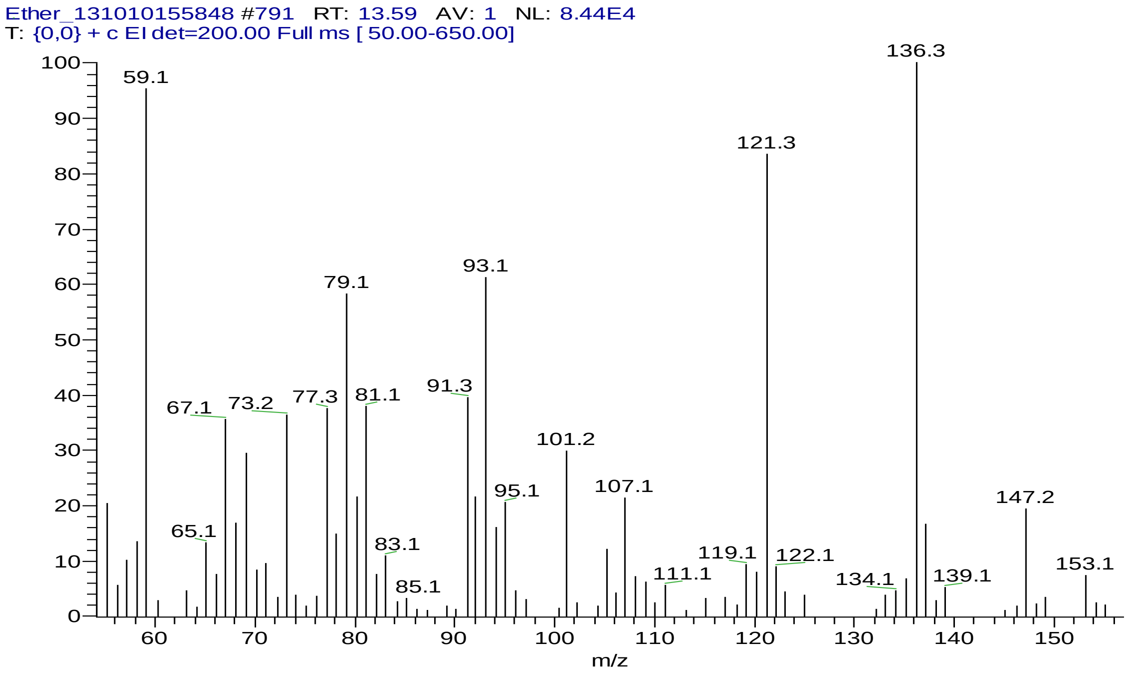

GC–MS spectrum

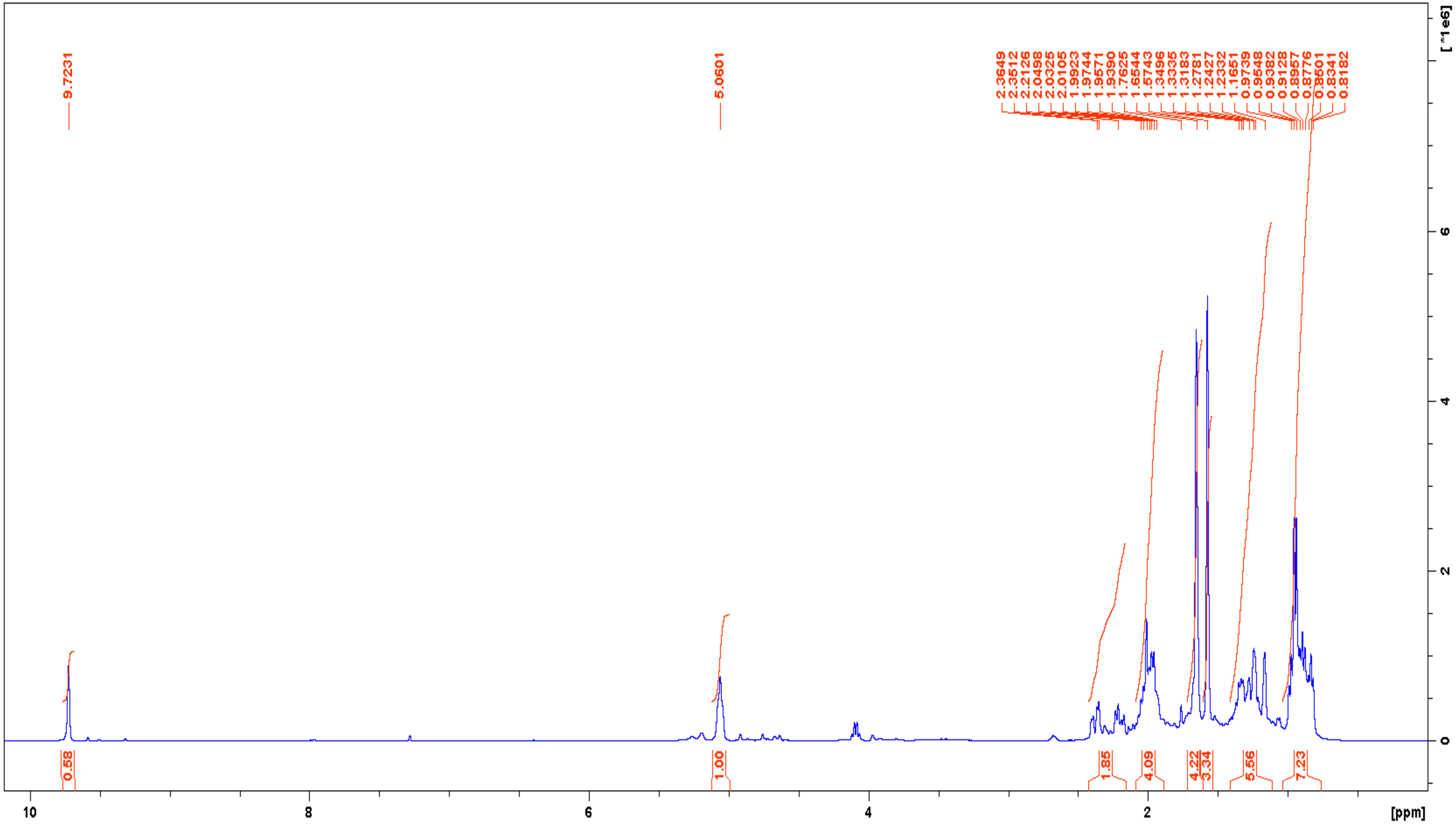

^{1}H NMR spectrum

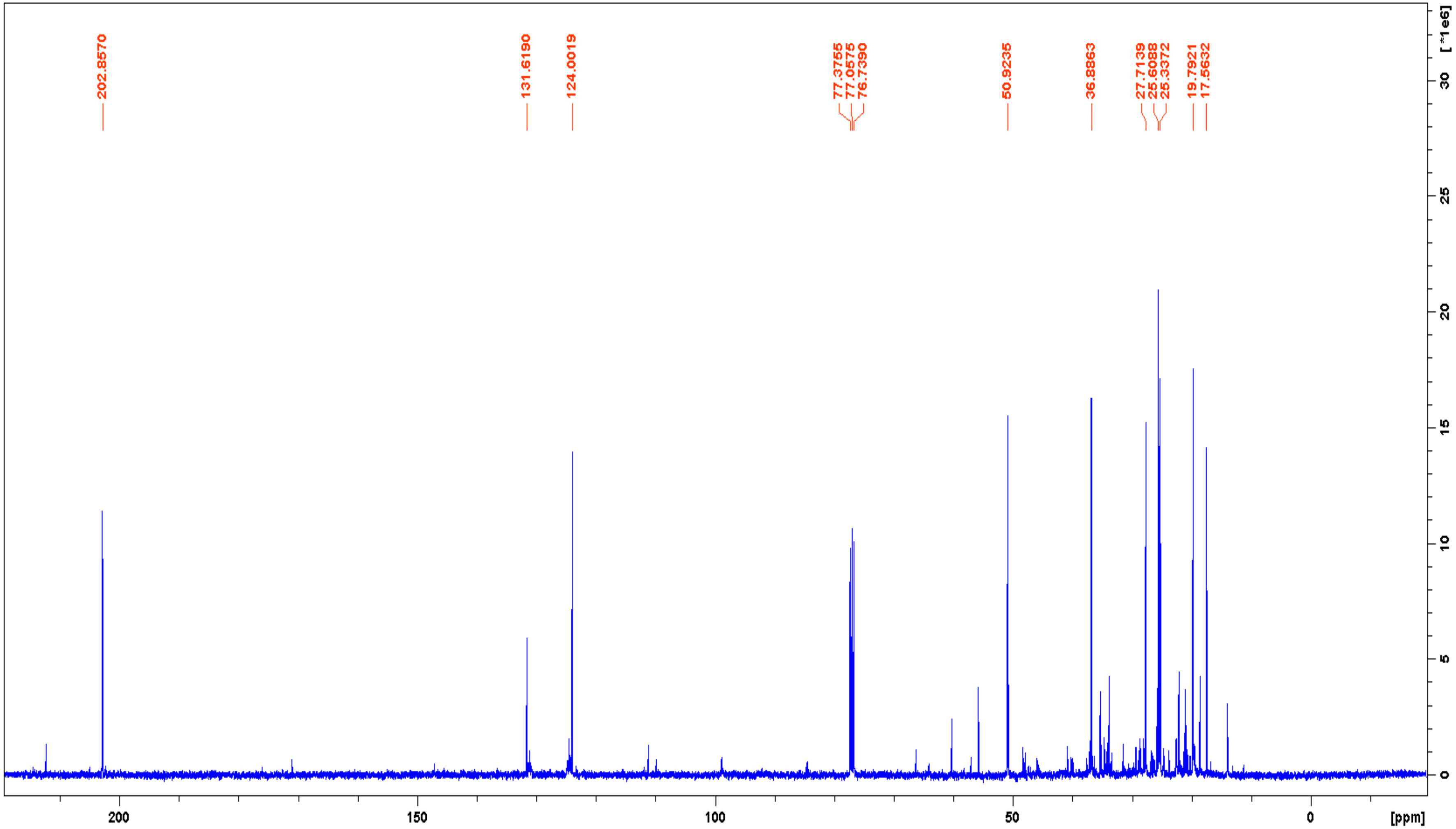

^{13}**C NMR spectrum**

1.3. *para*-menthane-3,8-diol

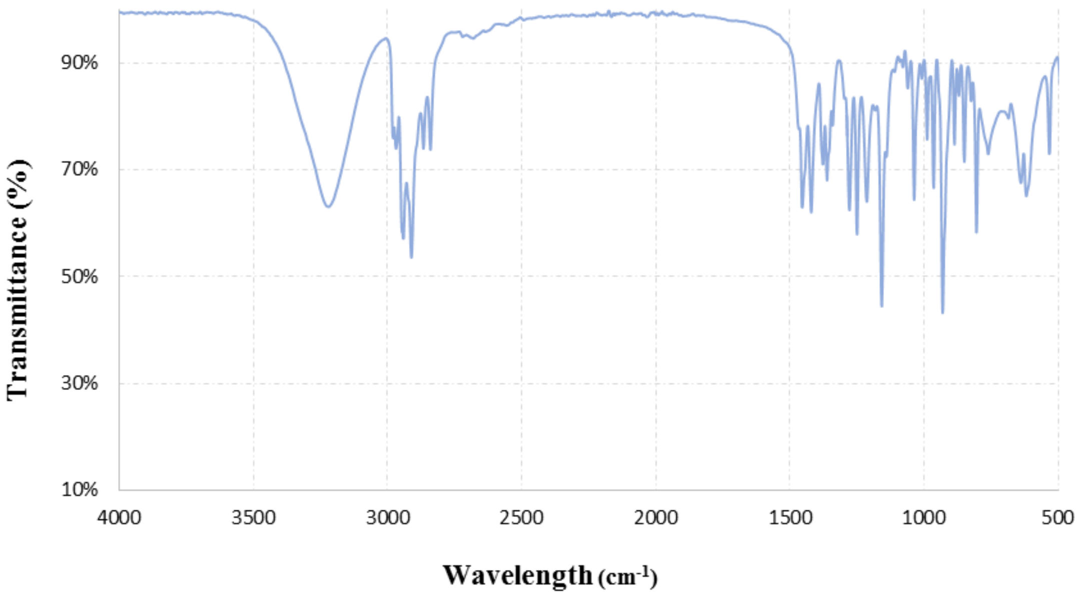

FTIR spectrum

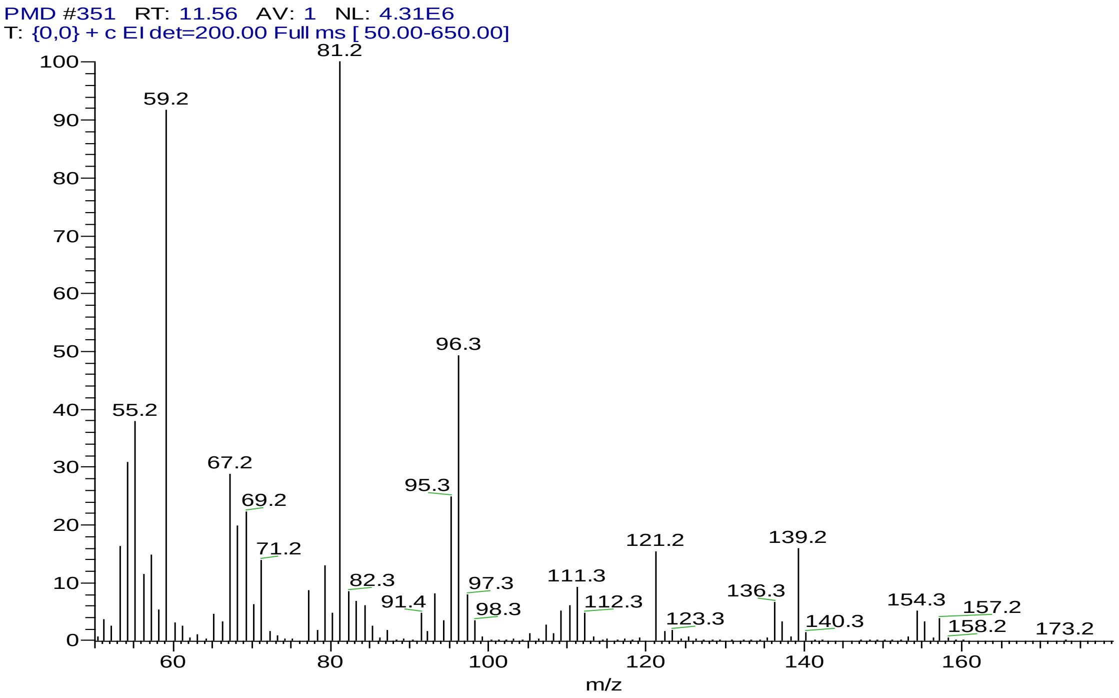

GC–MS spectrum

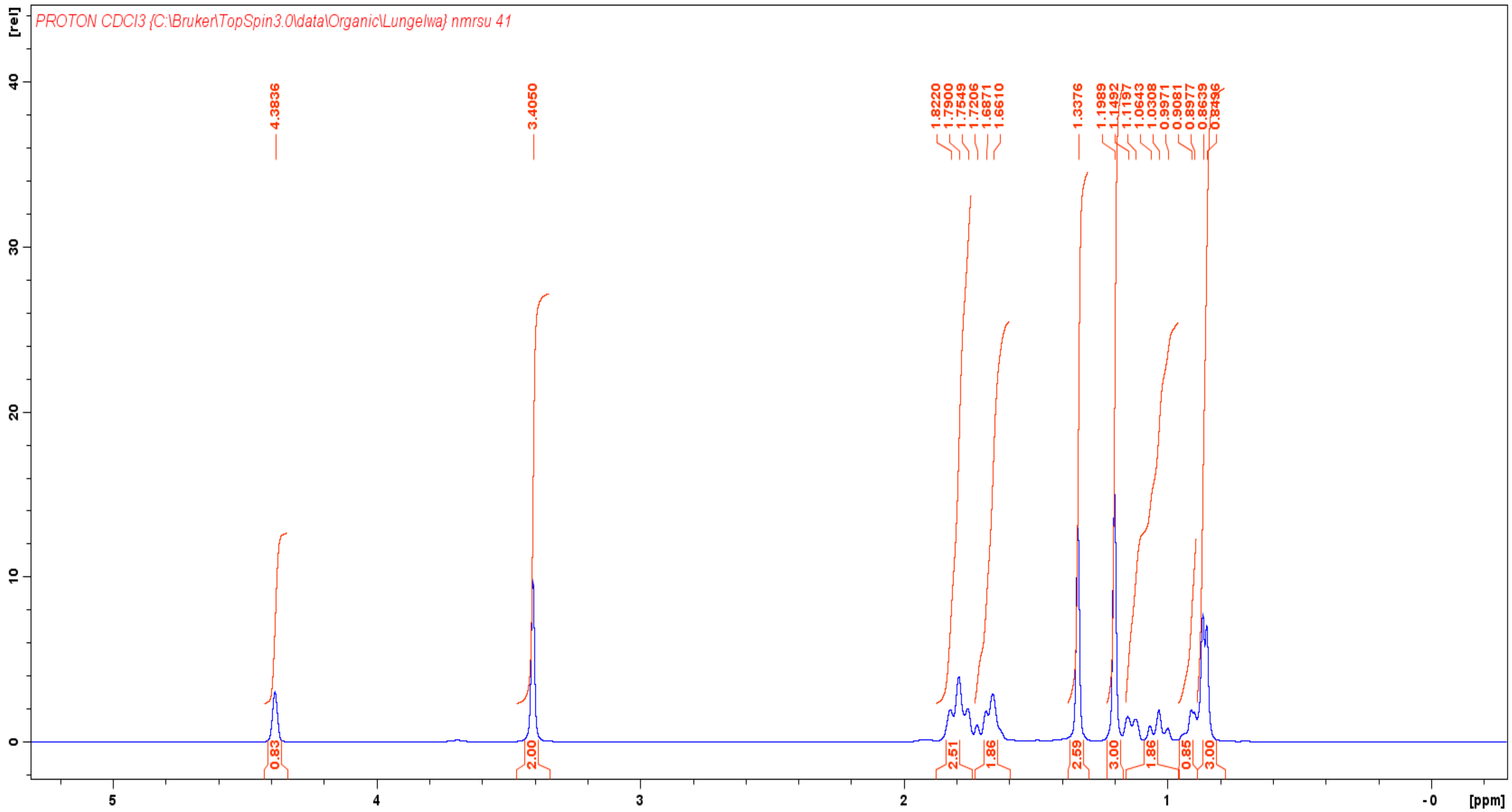

^{1}H NMR spectrum

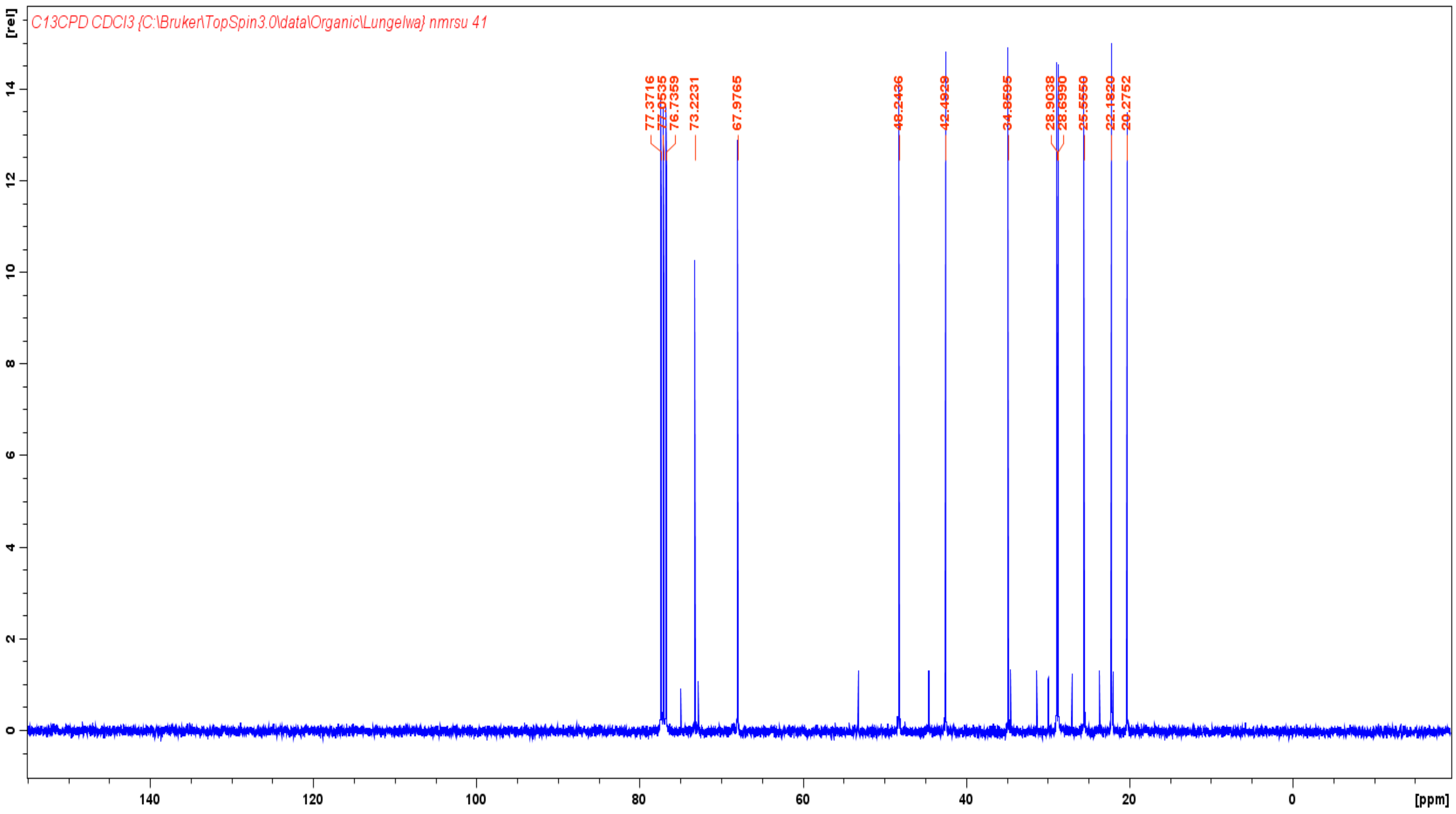

^{13}C NMR spectrum

Appendix B:

2. *para*-Menthane-3,8-diester derivatives

2.1. Mono-acetate

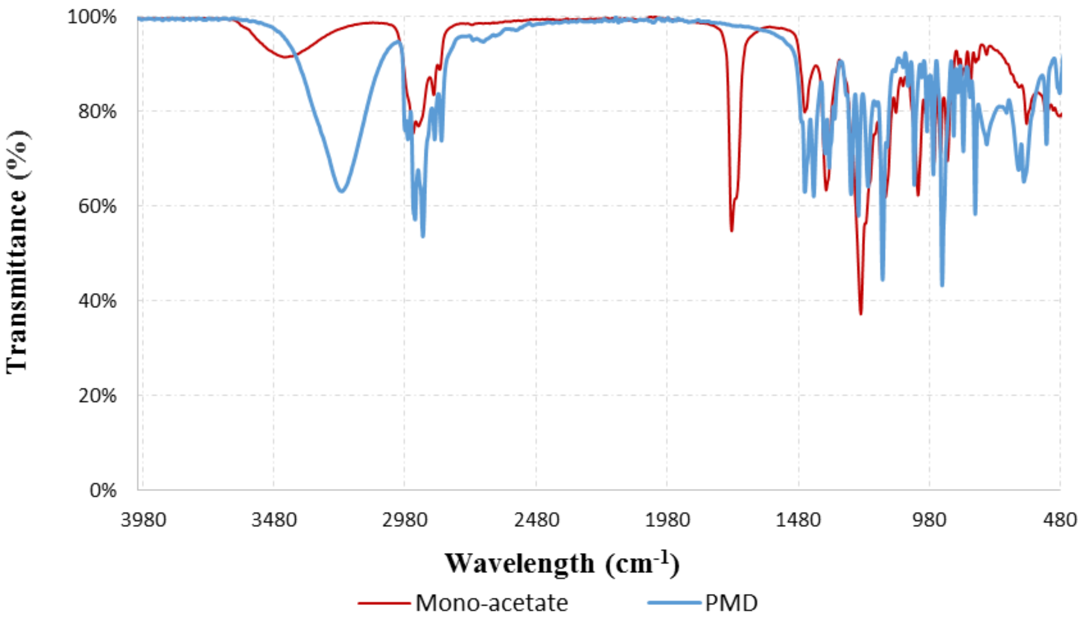

FTIR spectrum

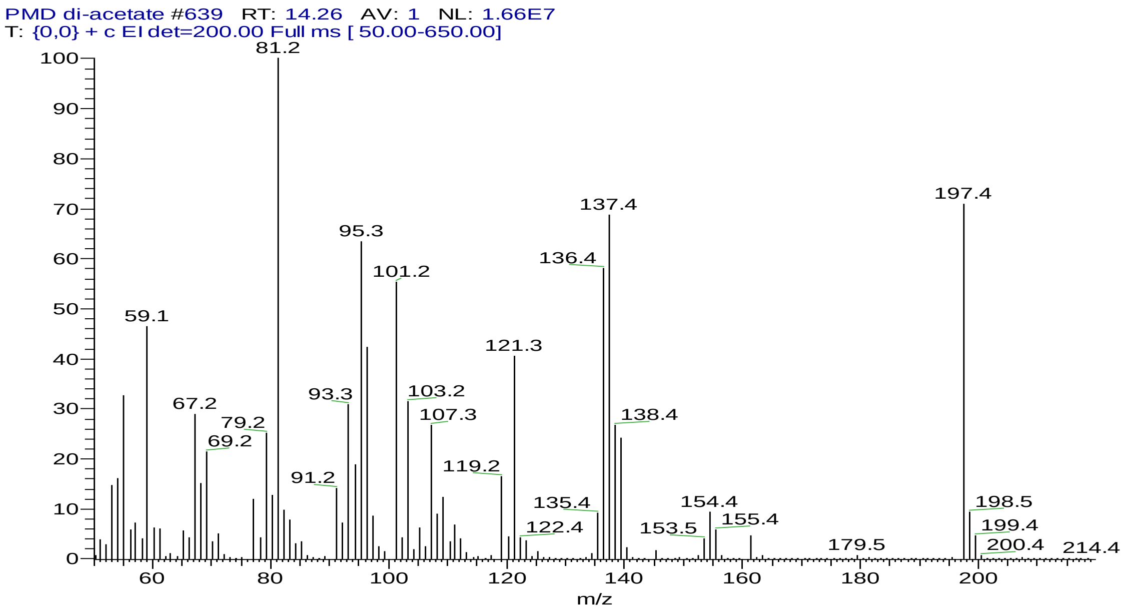

GC–MS spectrum

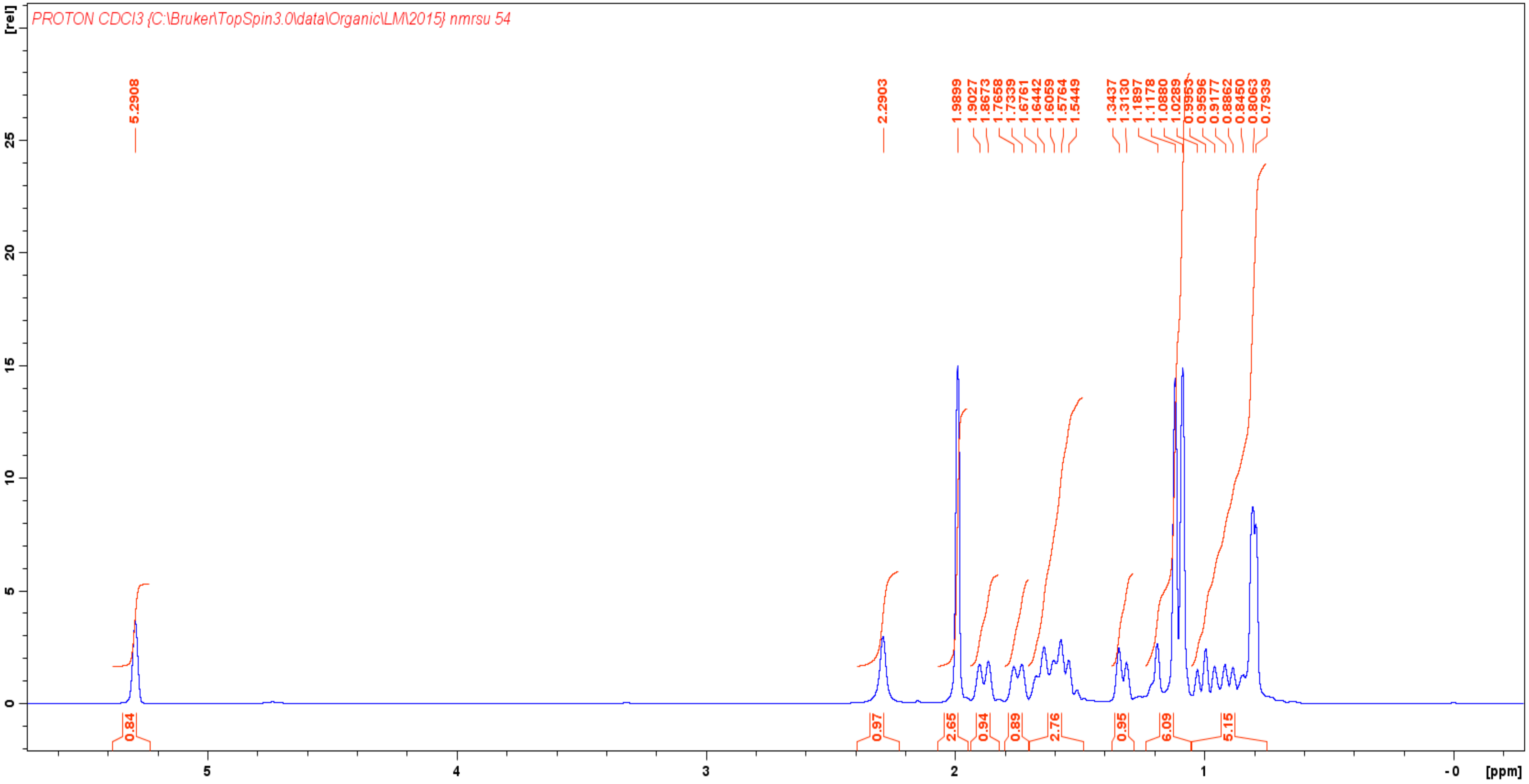

^{1}H NMR spectrum

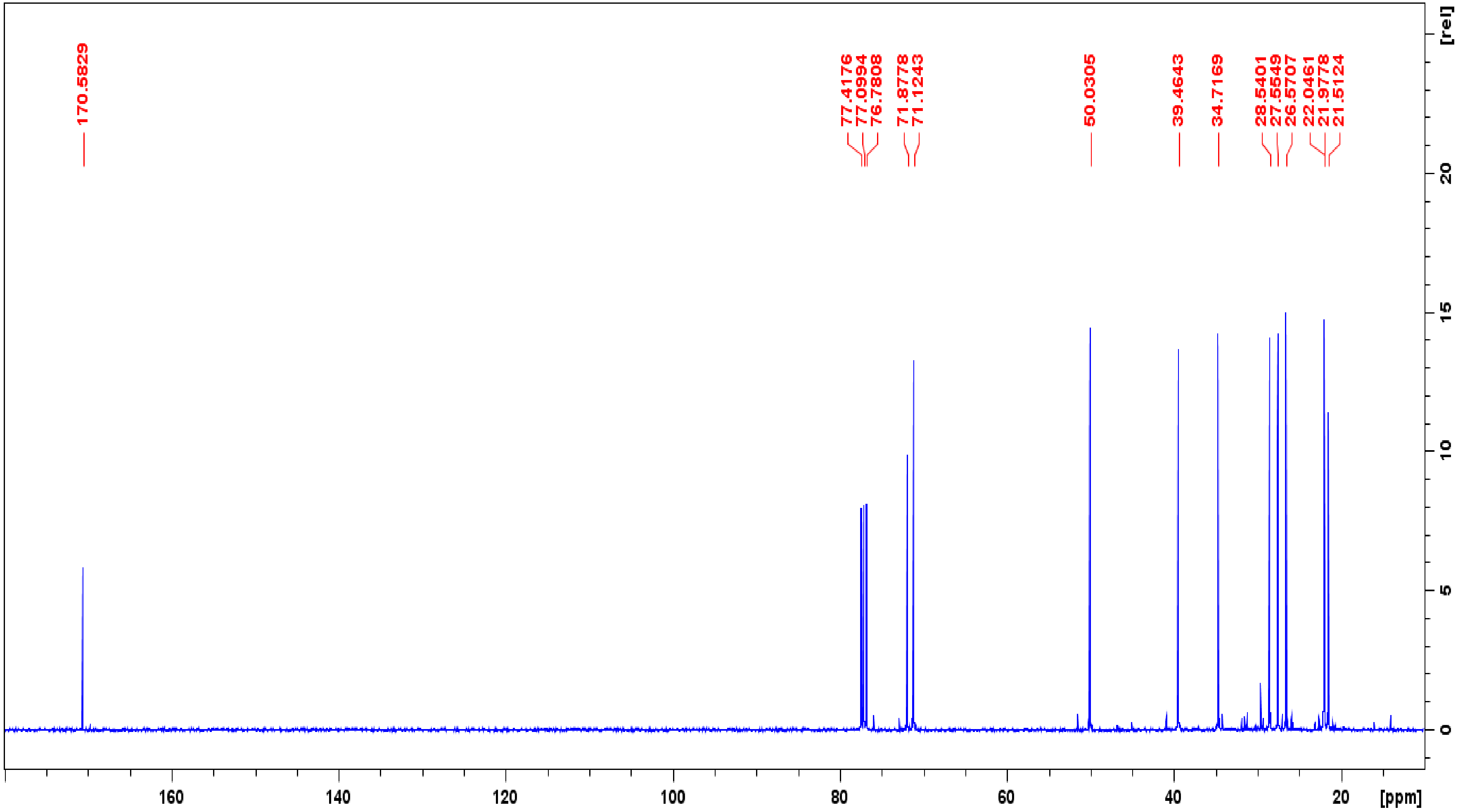

^{13}**C NMR spectrum**

2.2. Di-acetate

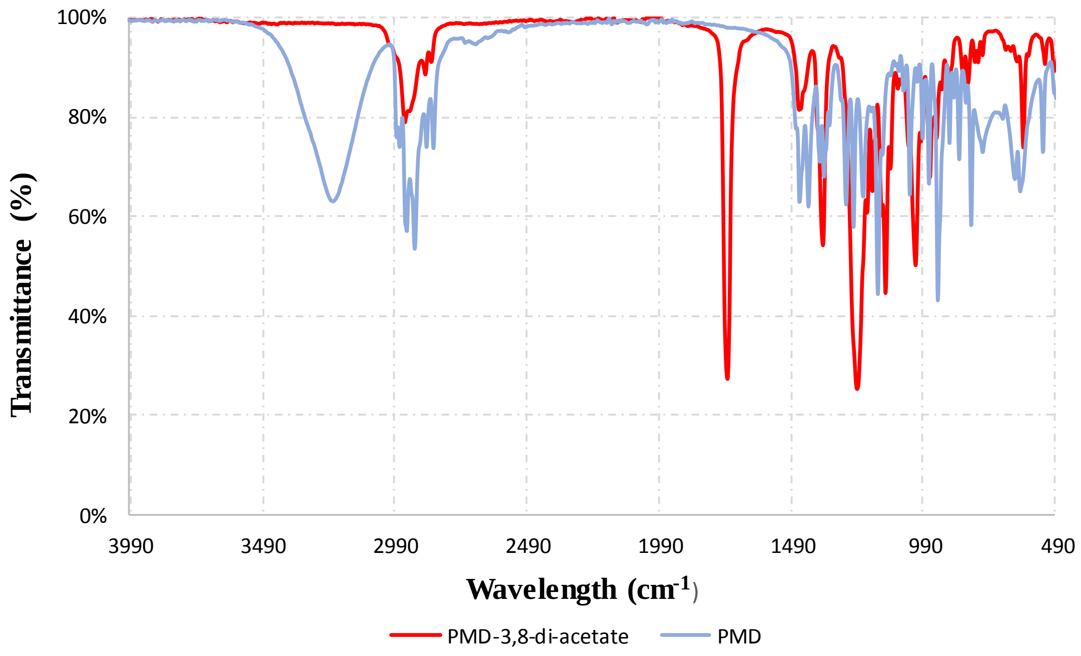

FTIR spectrum

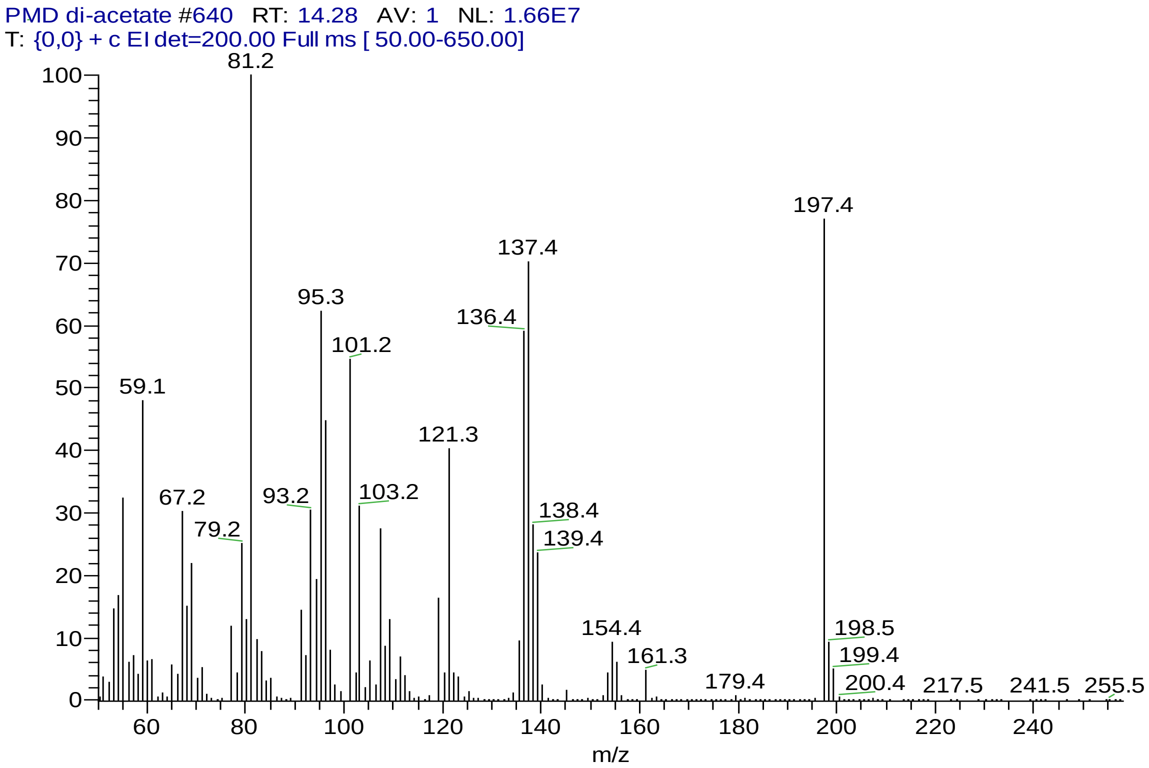

GC–MS spectrum

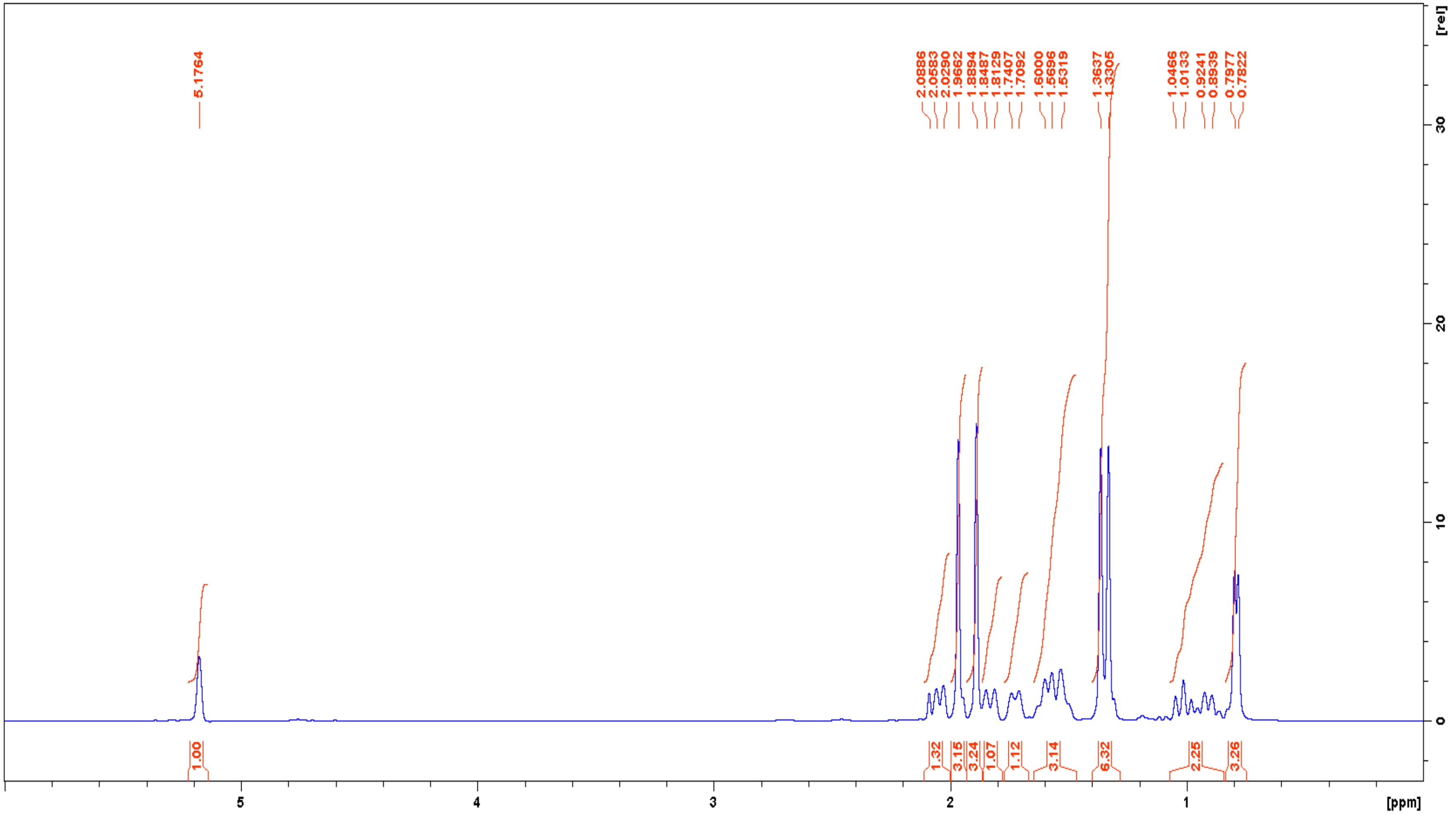

^{1}H NMR spectrum

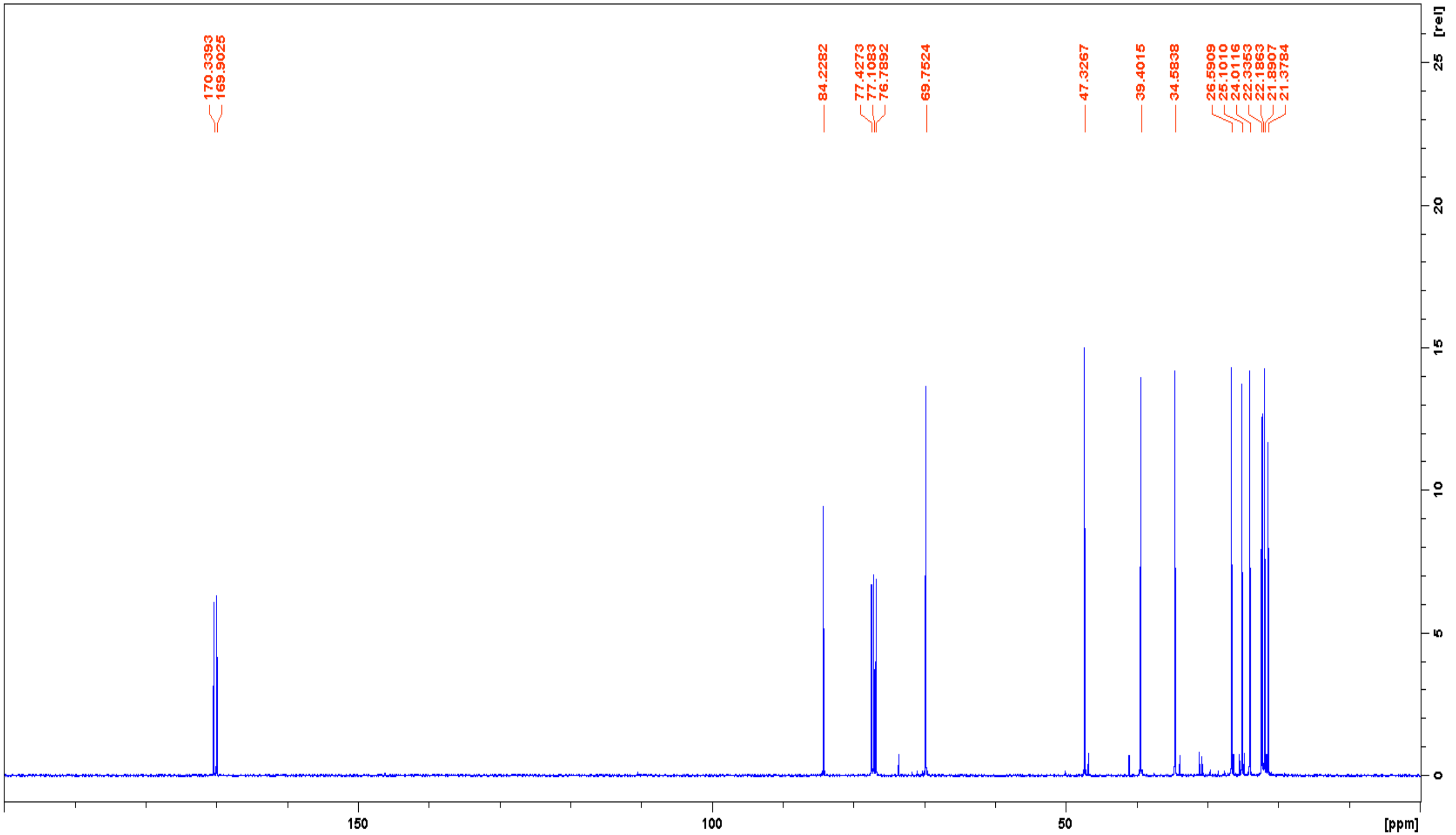

^{13}**C NMR spectrum**

2.3. Mono-propionate

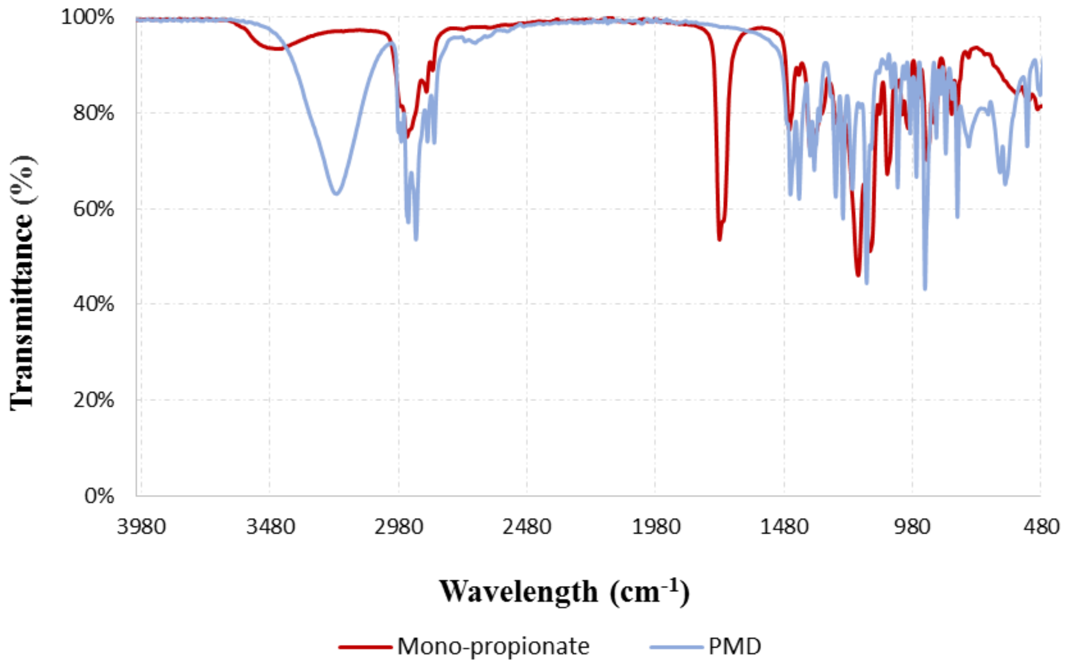

FTIR spectrum

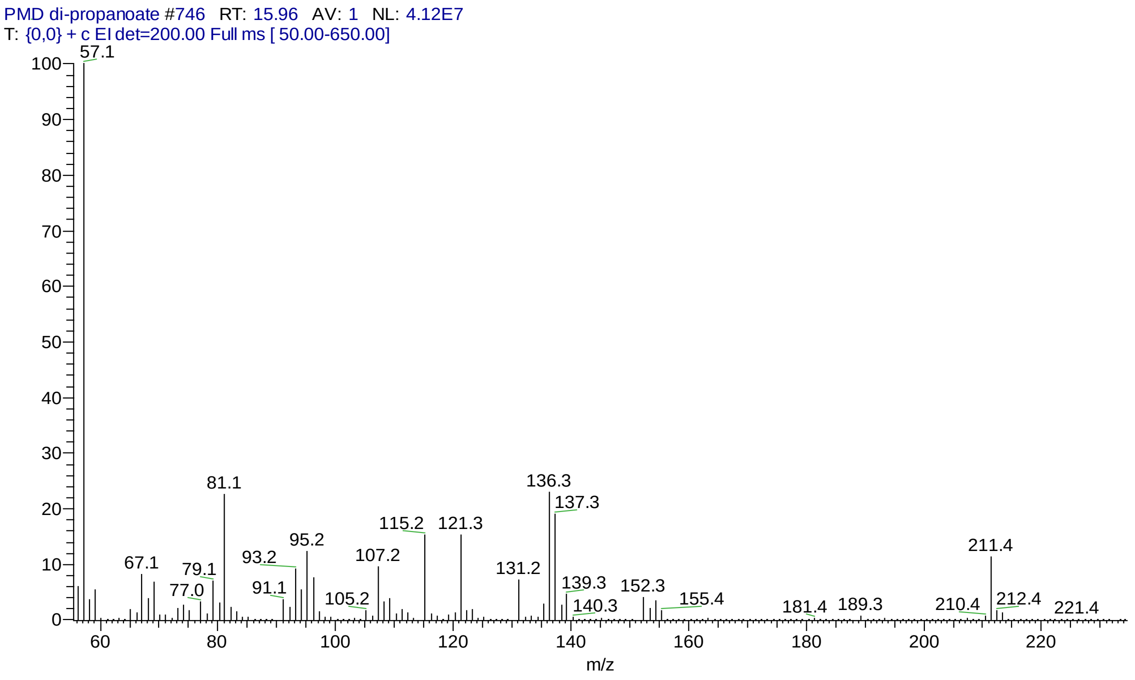

GC–MS spectrum

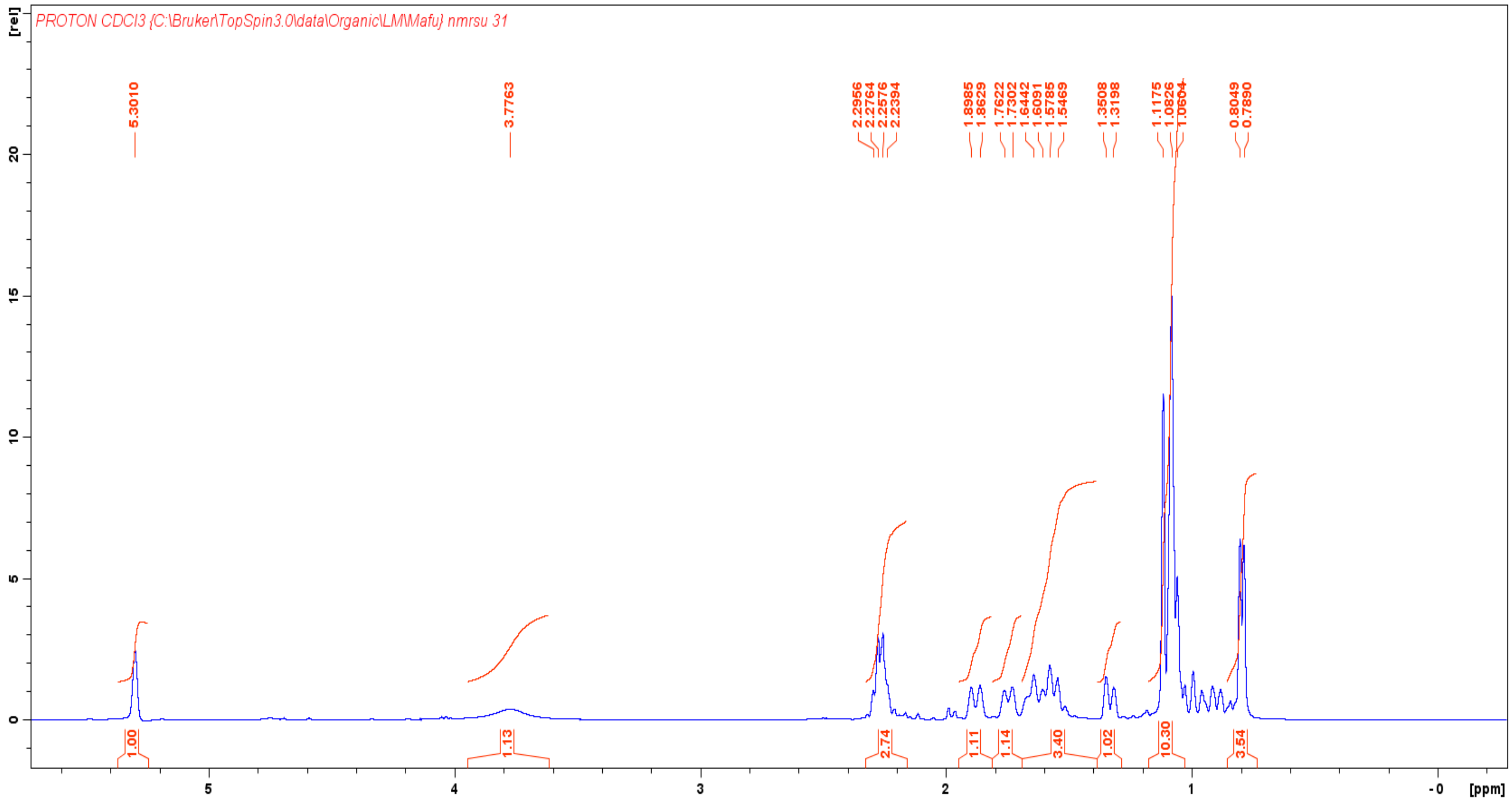

^{1}H NMR spectrum

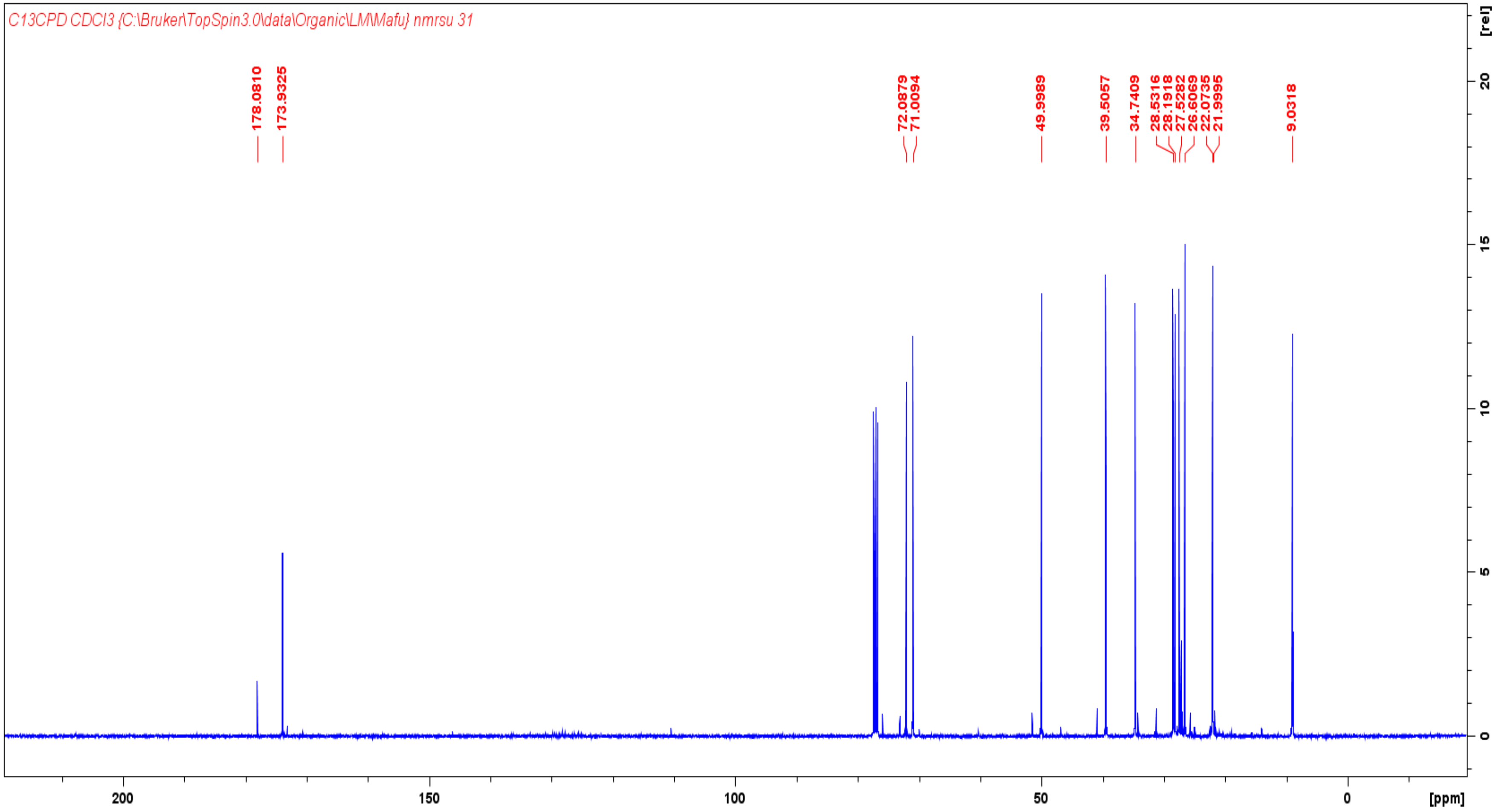

^{13}C NMR spectrum

2.4. Di-propionate

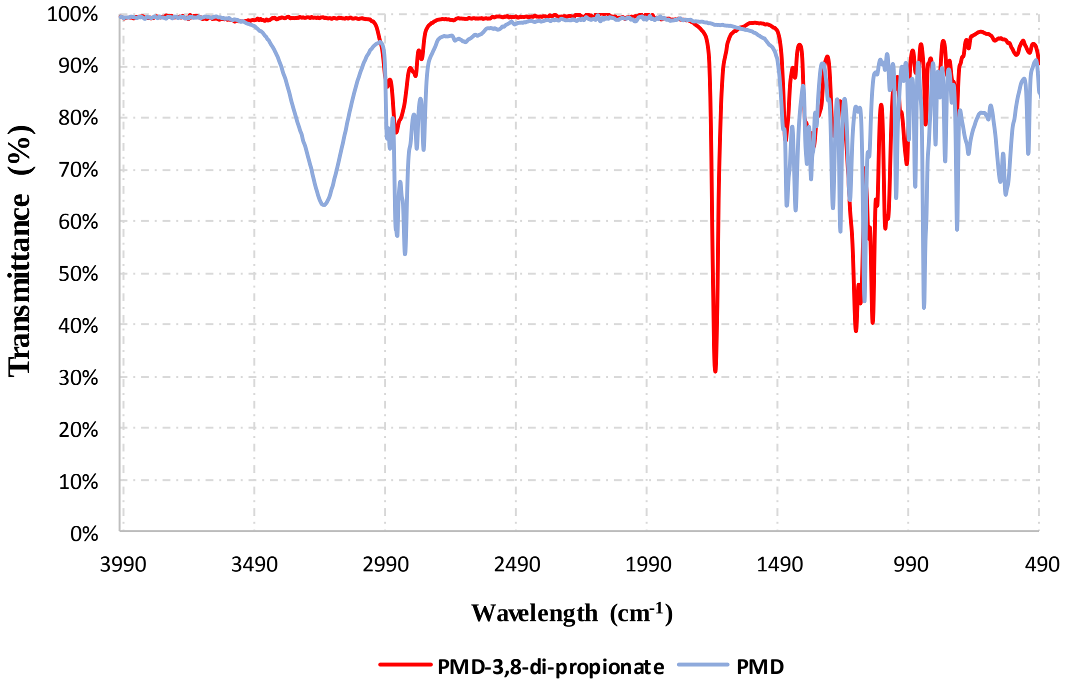

FTIR spectrum

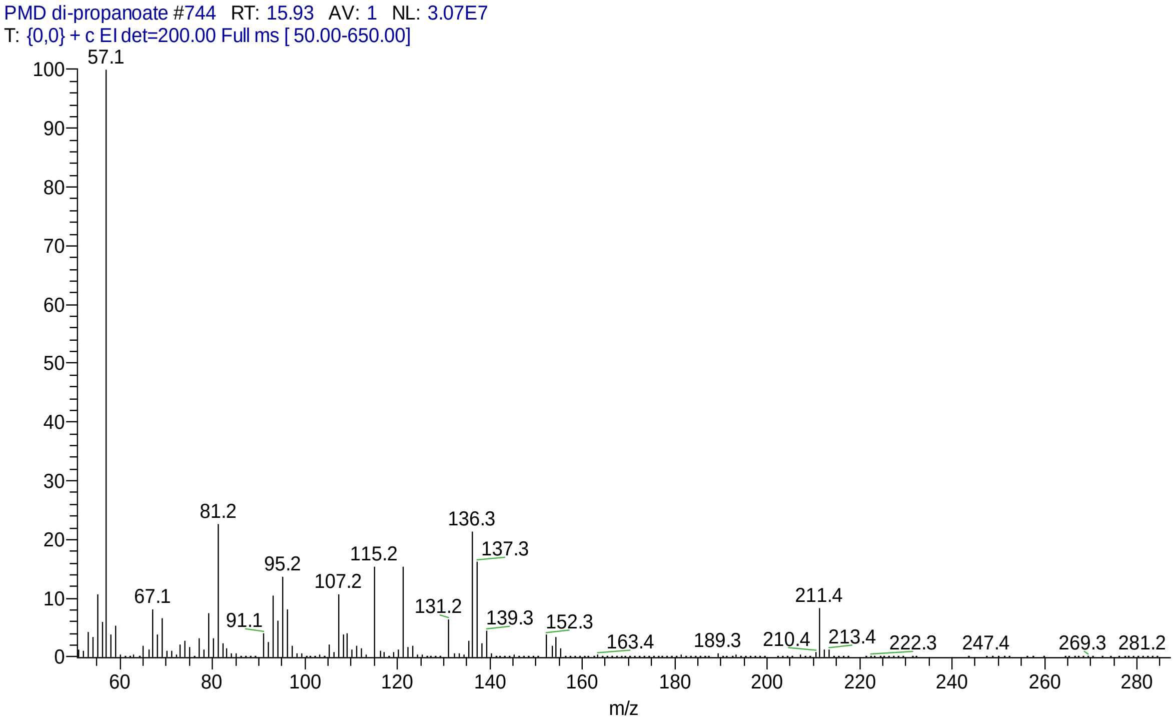

GC–MS spectrum

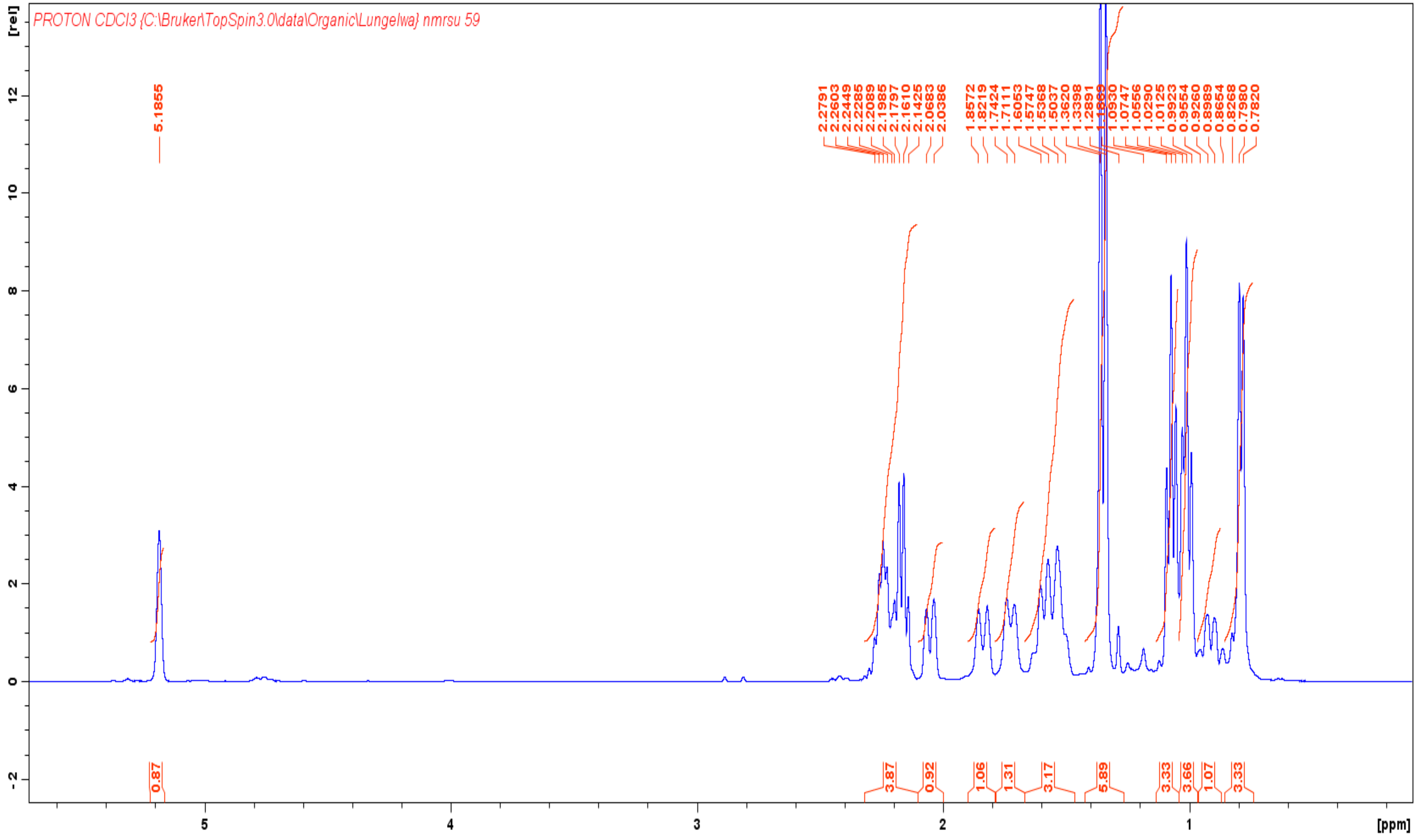

^{1}H NMR spectrum

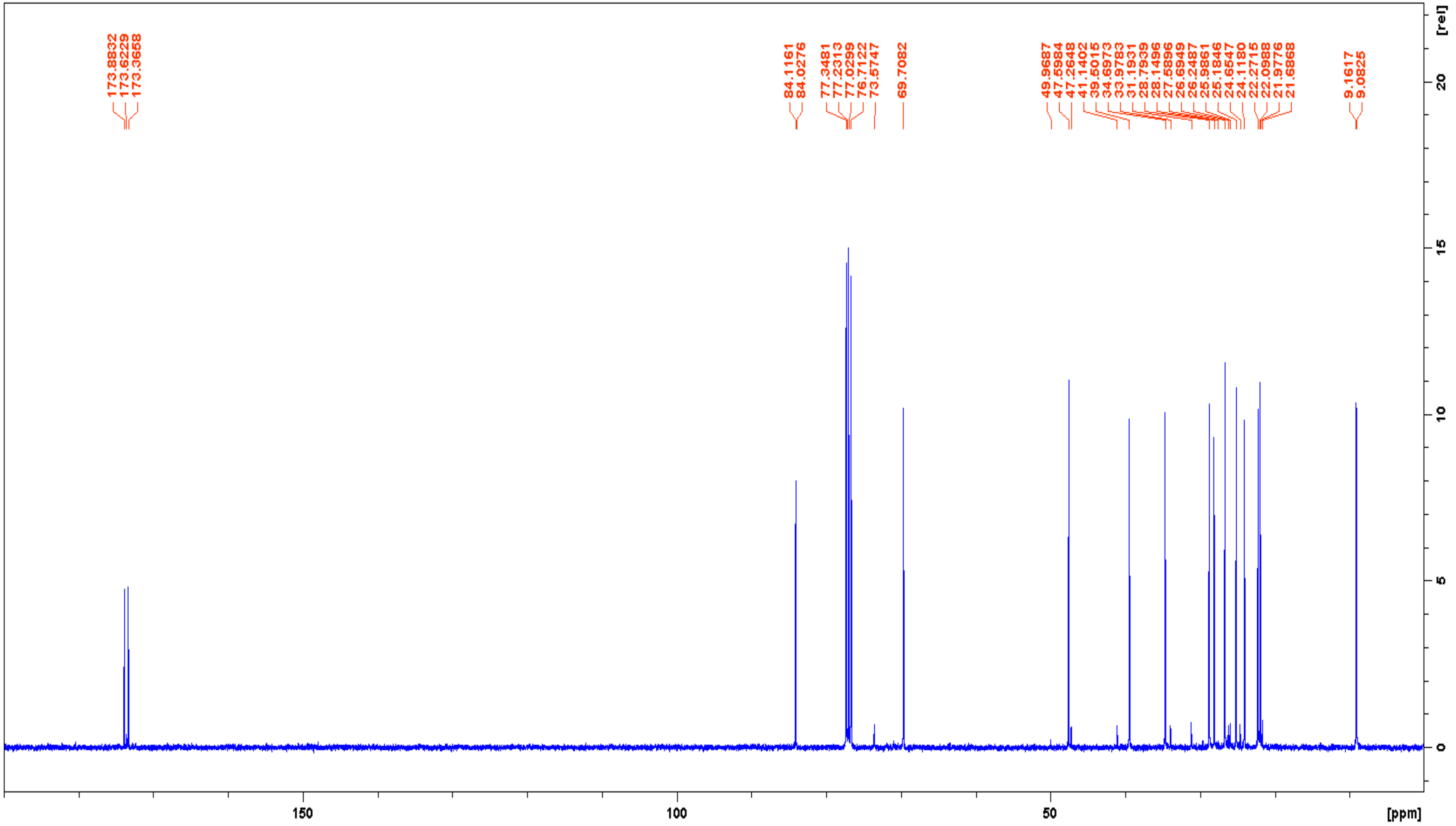

^{13}C NMR spectrum

2.5. Mono-pentanoate

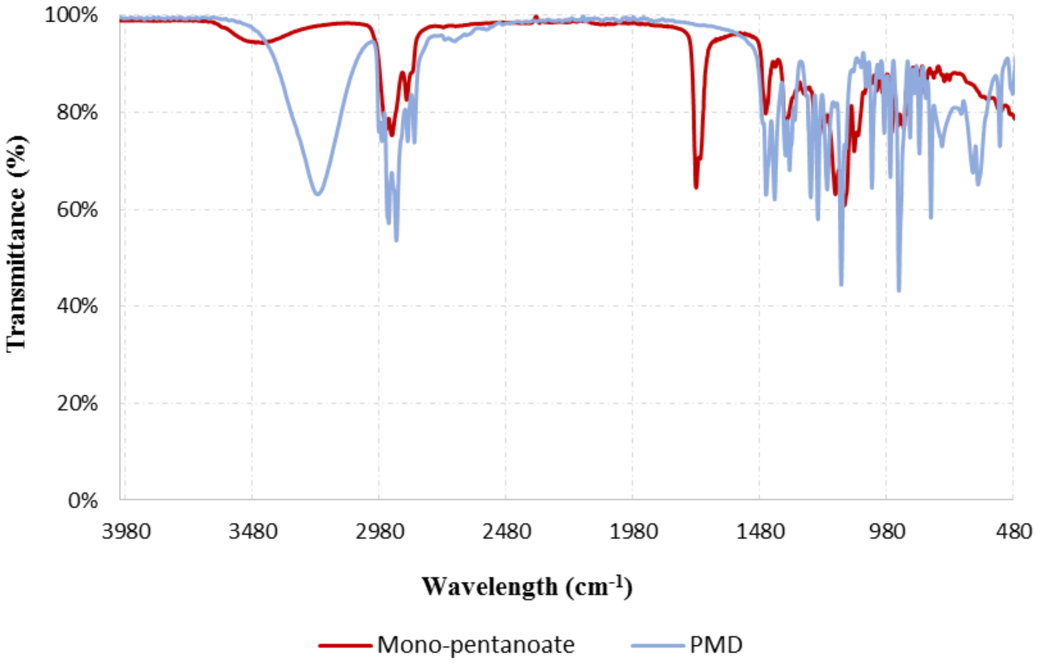

FTIR spectrum

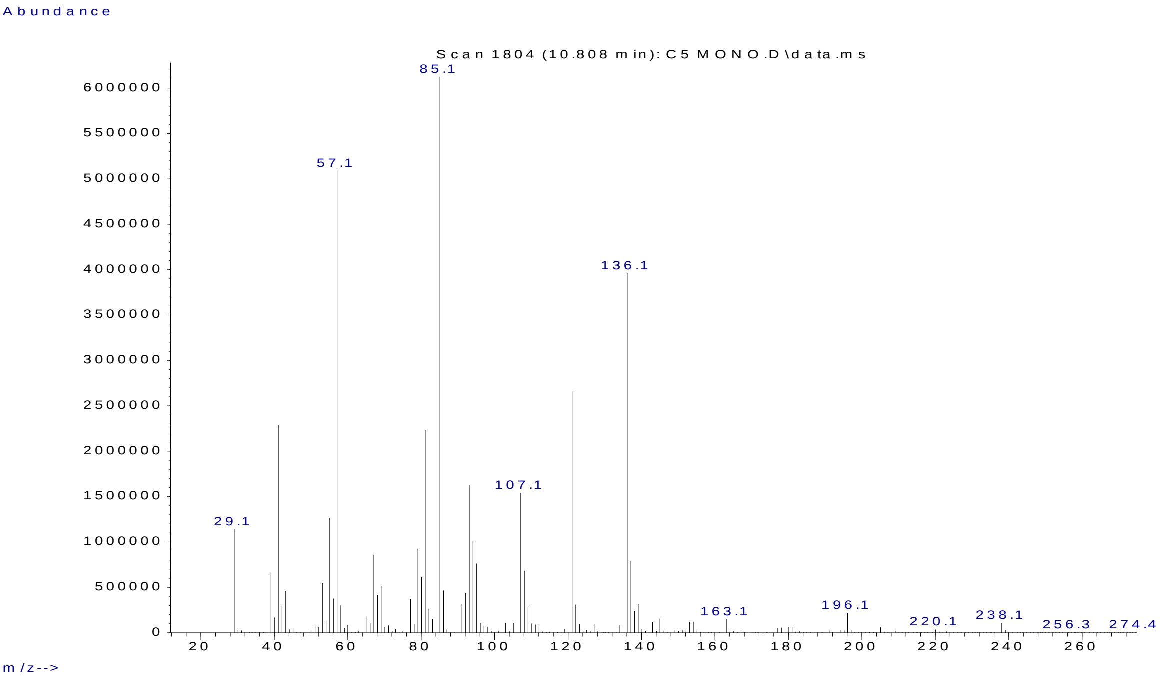

GC–MS spectrum

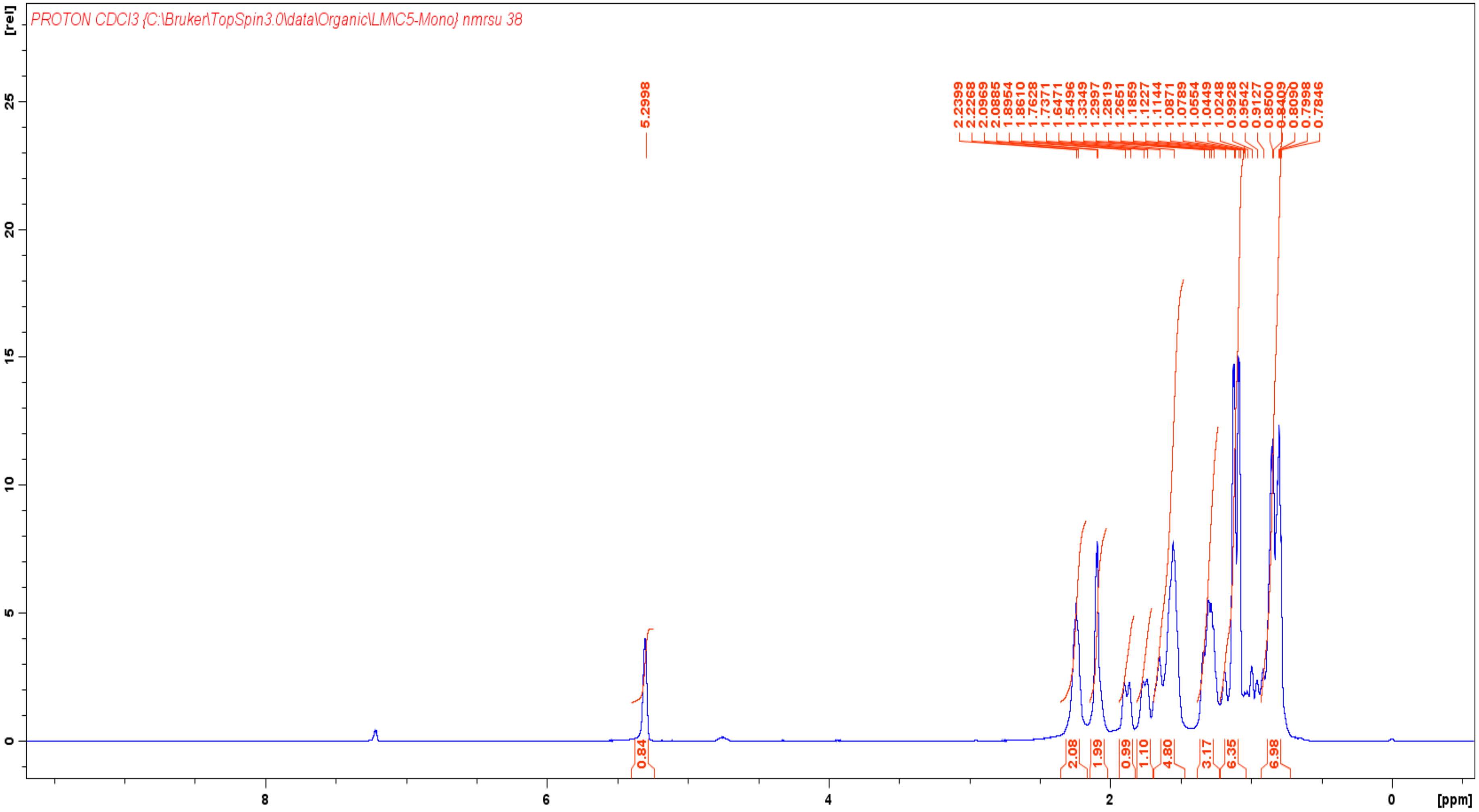

^{1}H NMR spectrum

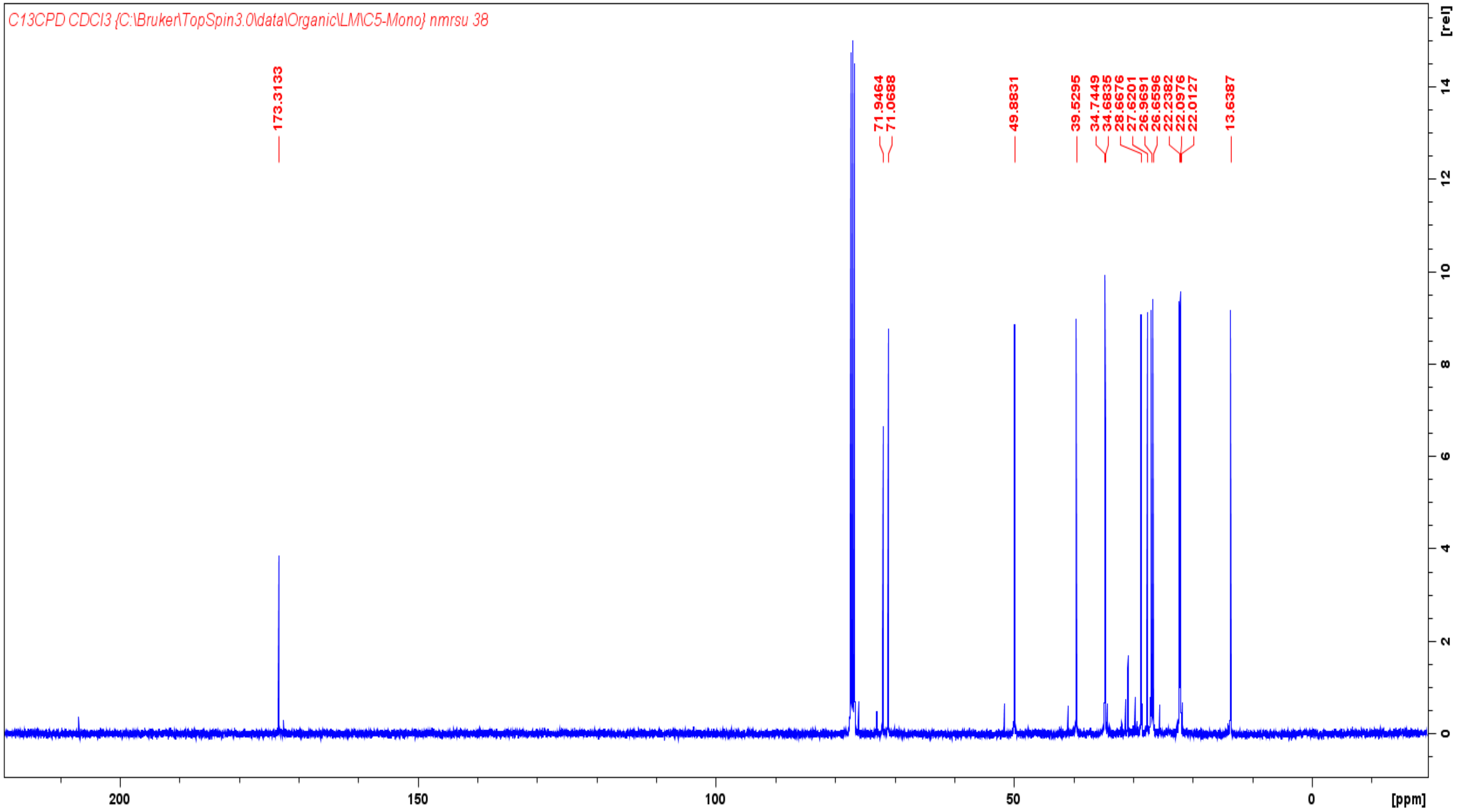

^{13}C NMR spectrum

2.6. Di-pentanoate

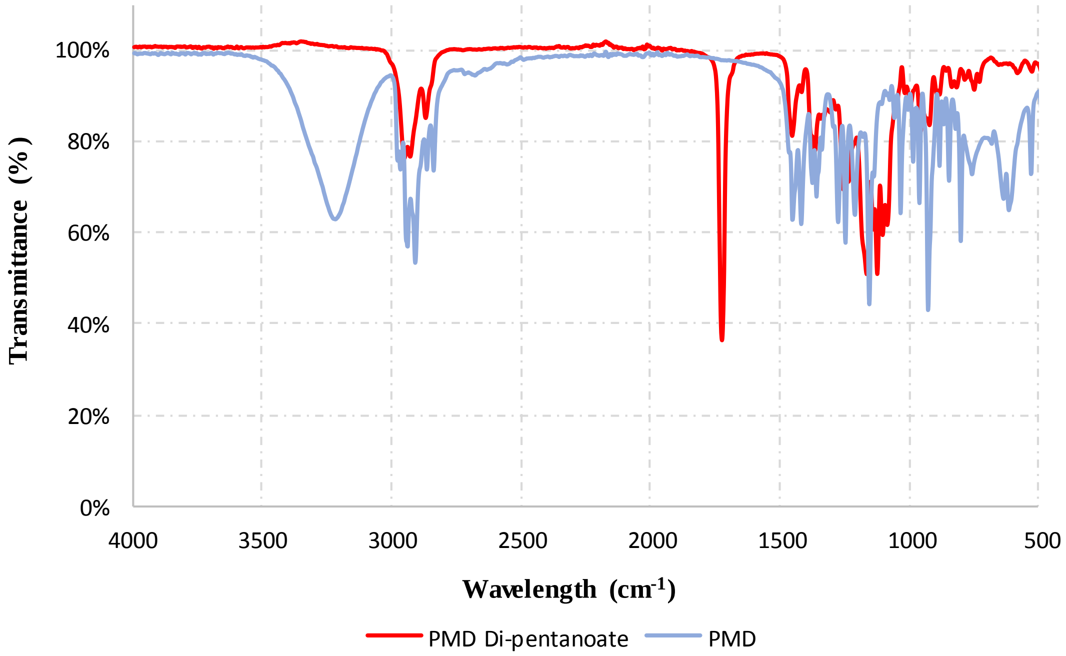

FTIR spectrum

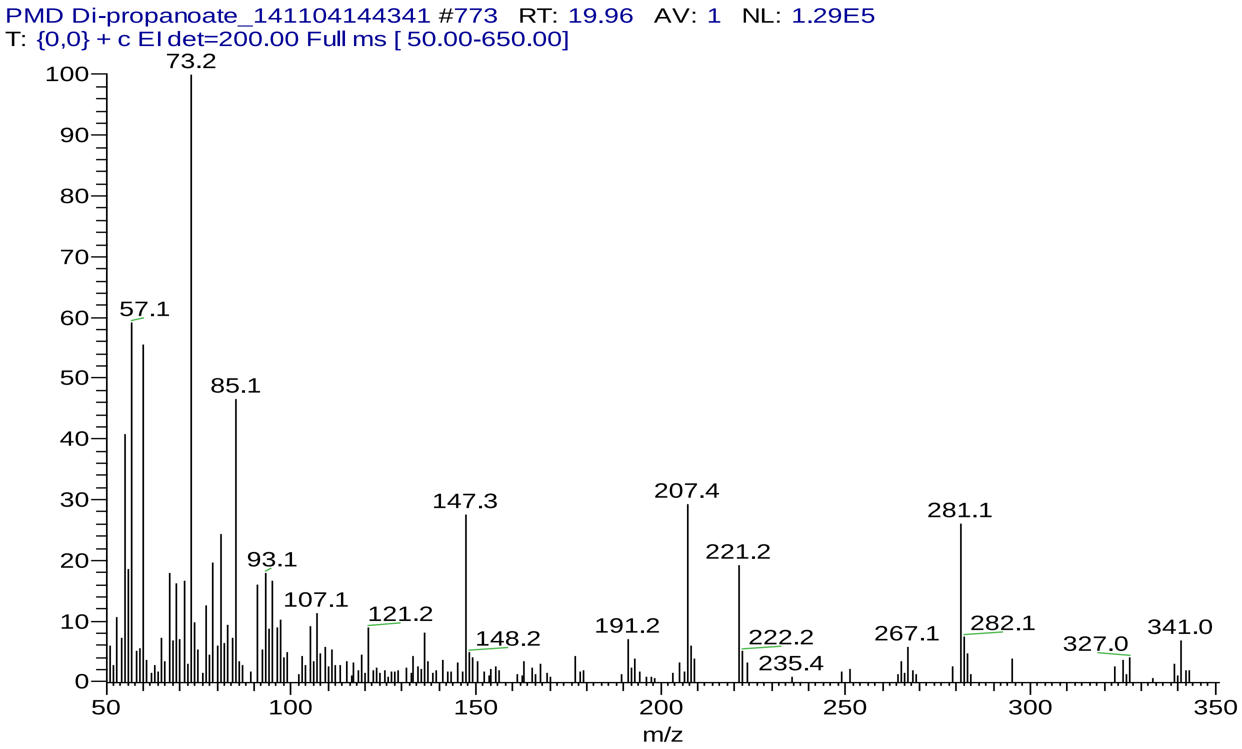

GC–MS spectrum

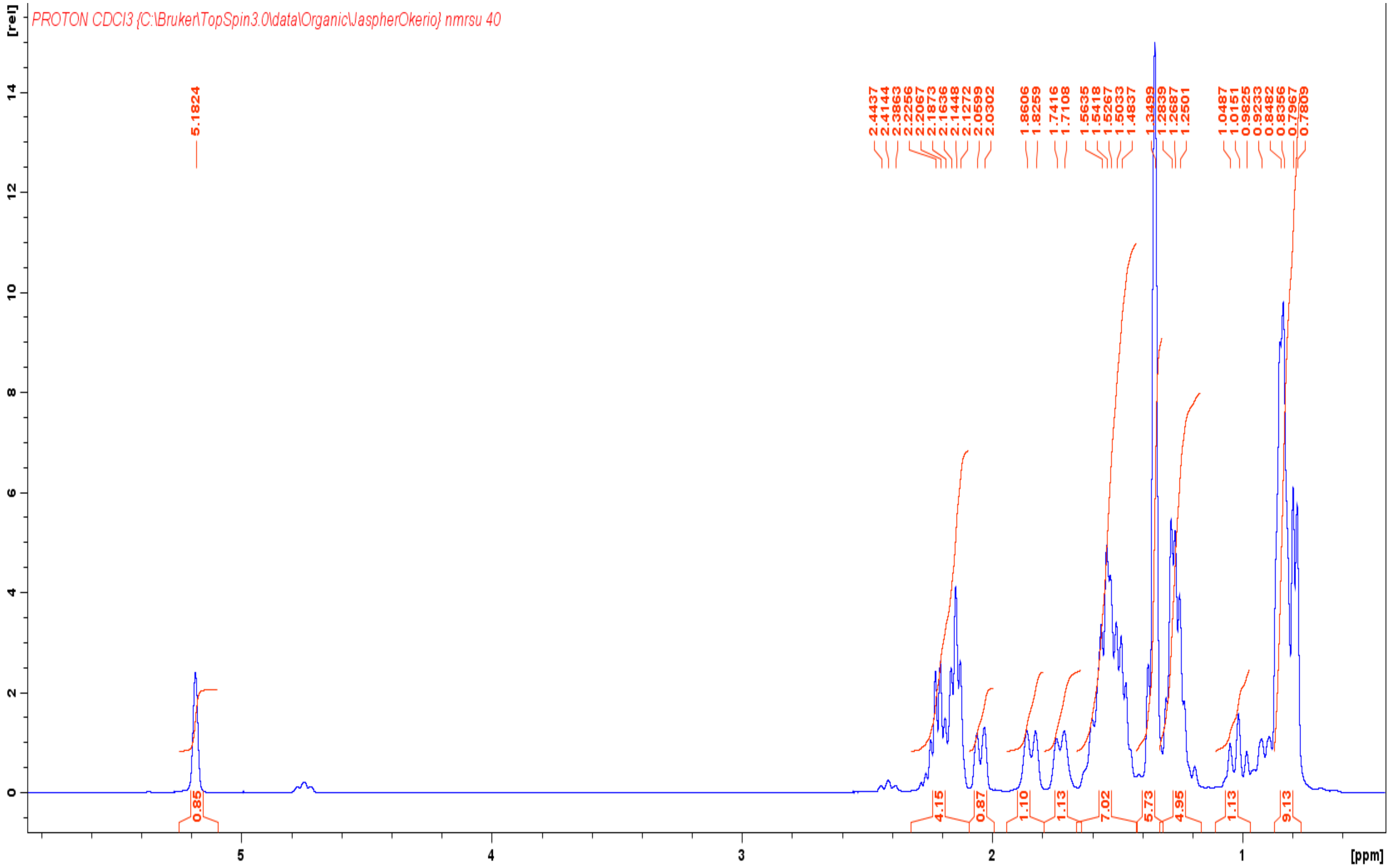

^{1}H NMR spectrum

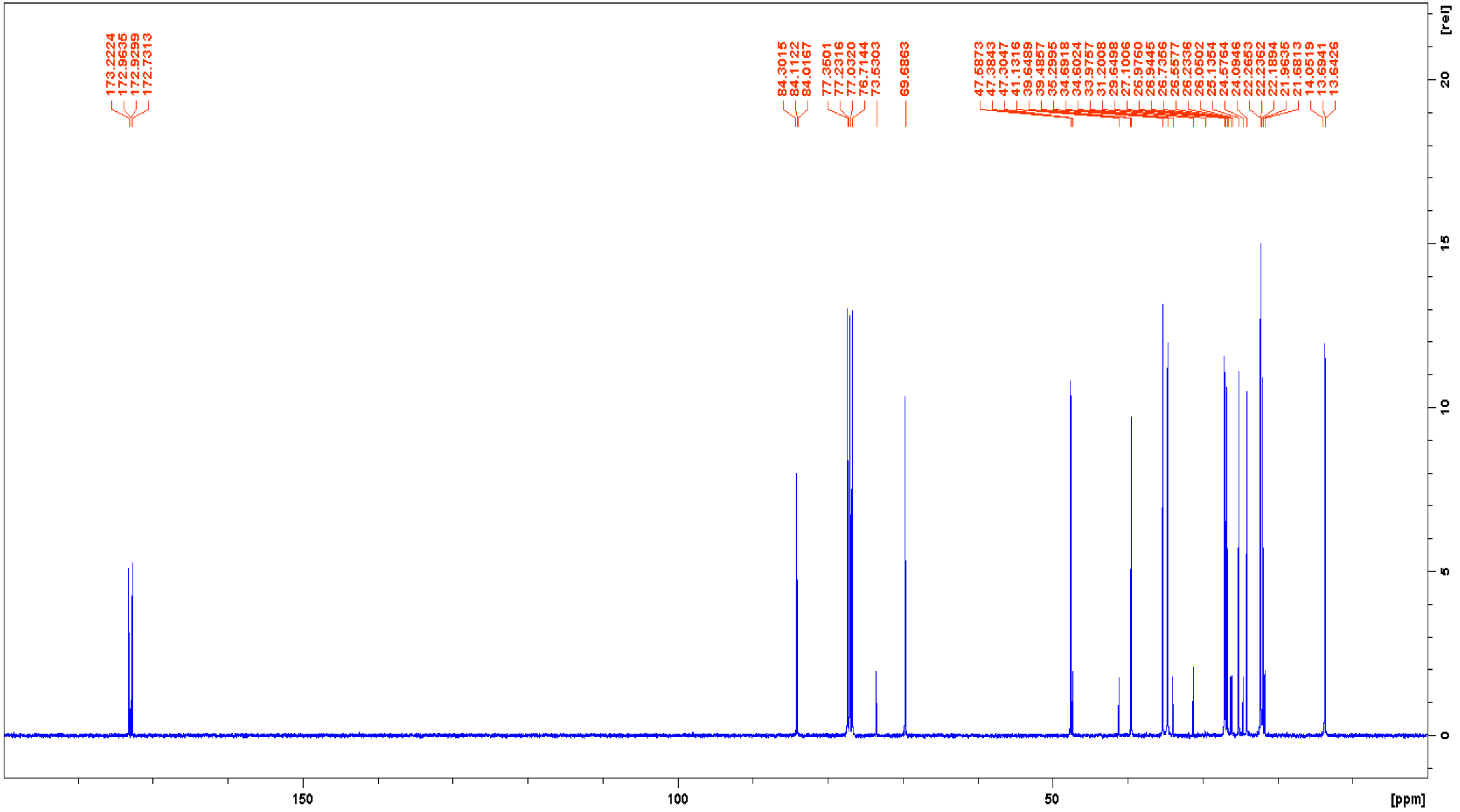

^{13}C NMR spectrum

2.7. Mono-hexanoate

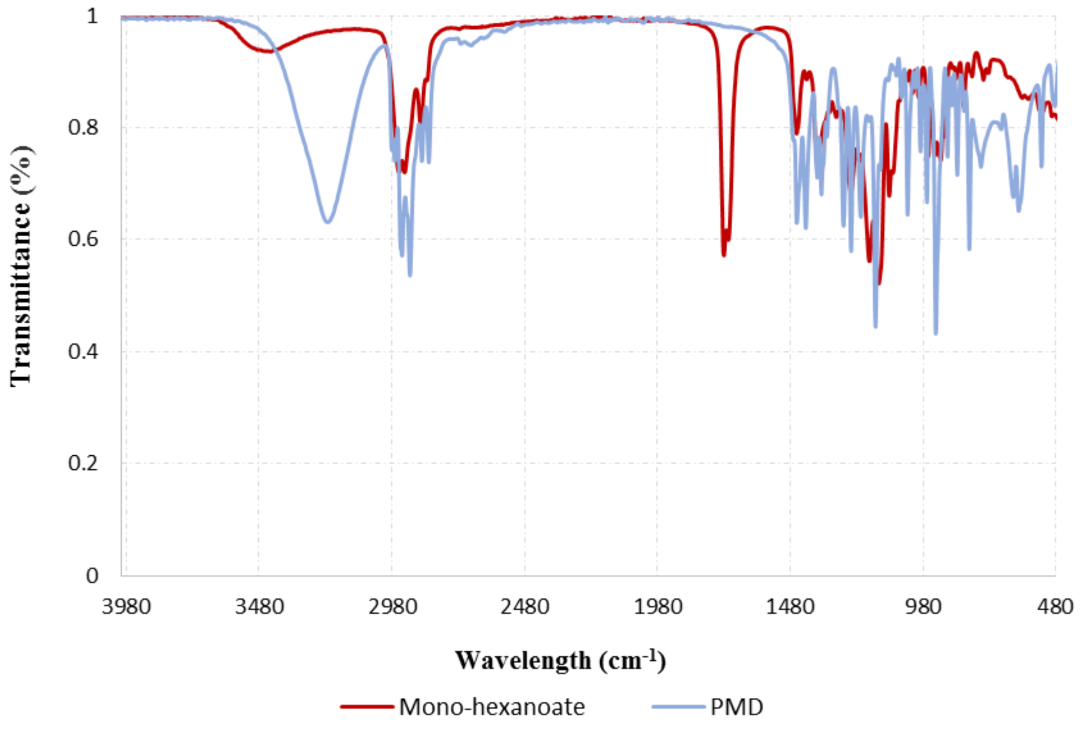

FTIR spectrum

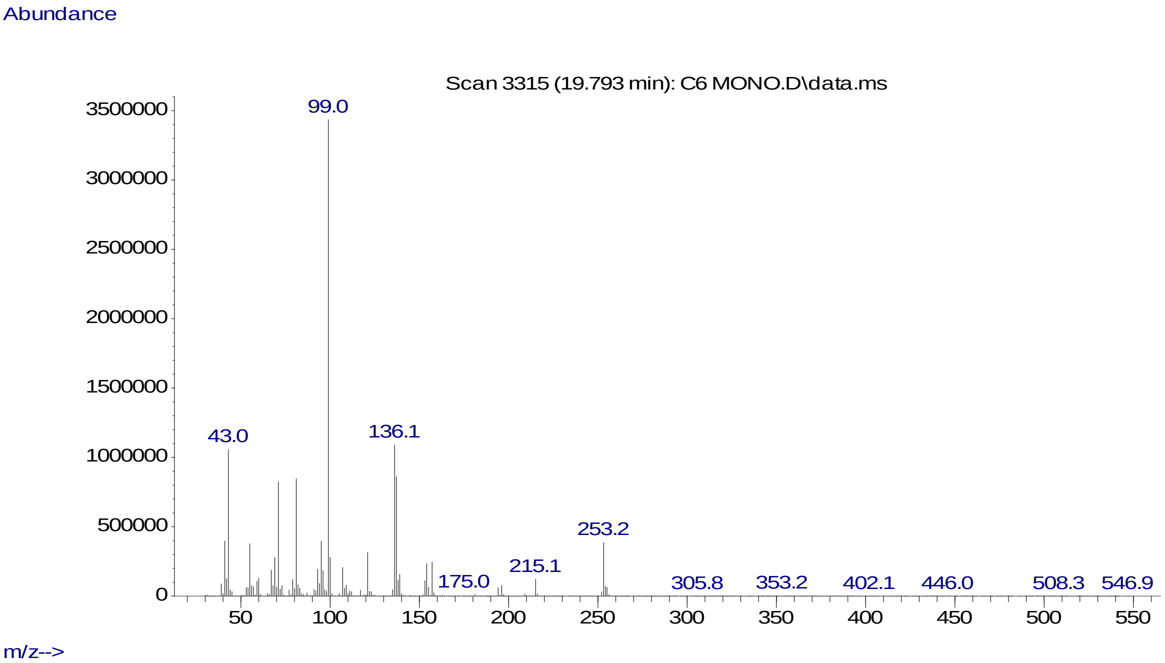

GC–MS spectrum

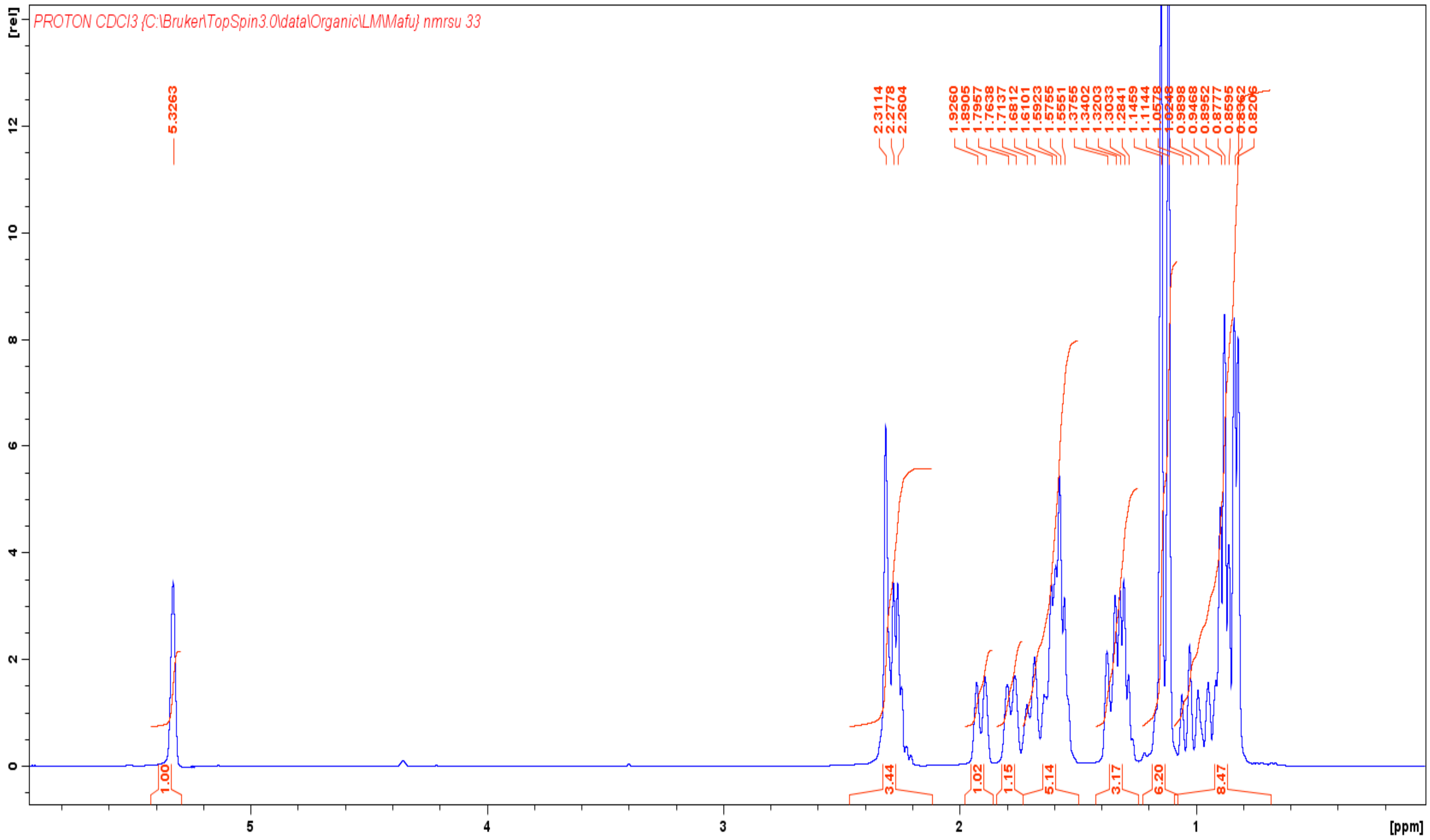

^{1}H NMR spectrum

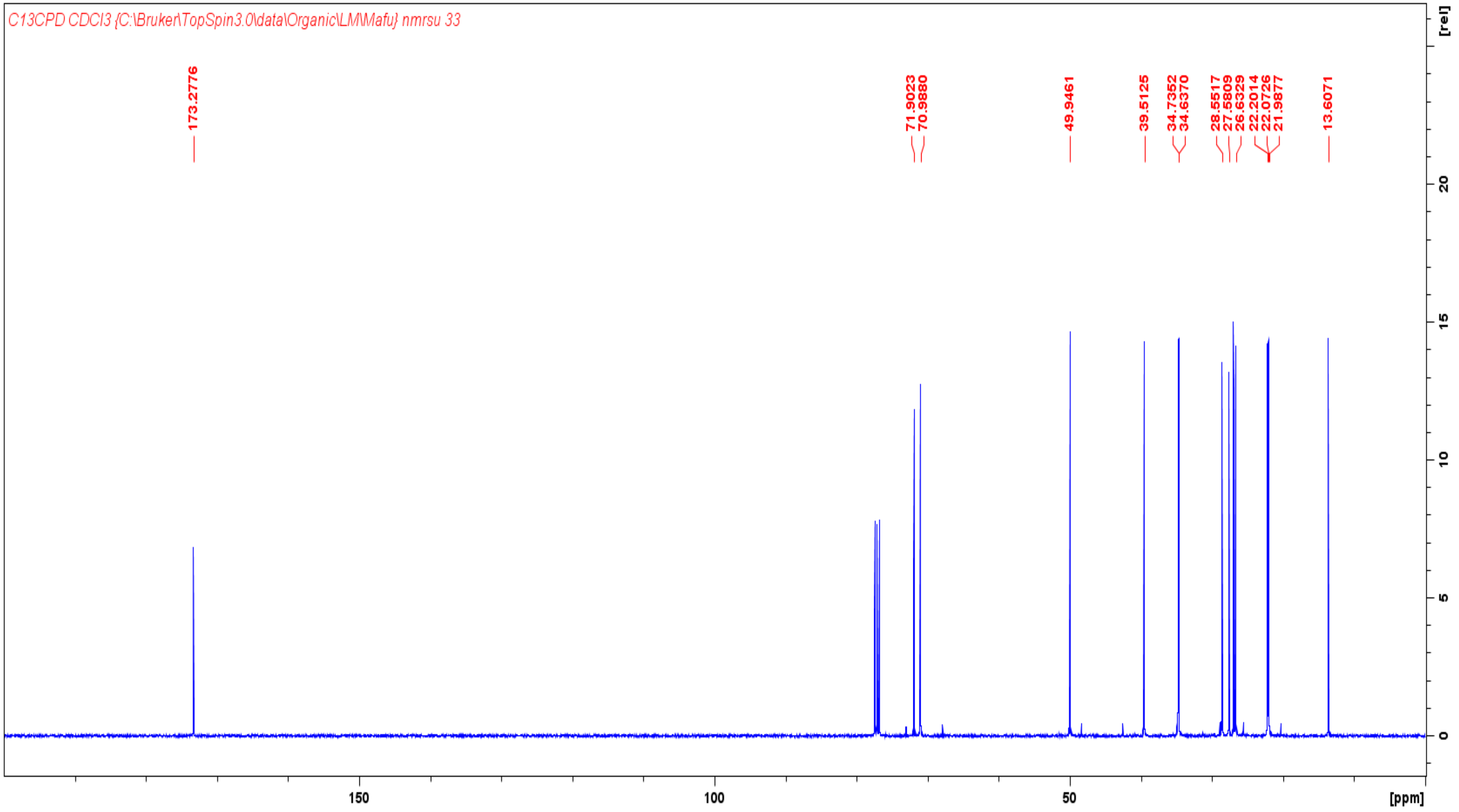

^{13}C NMR spectrum

Di-hexanoate

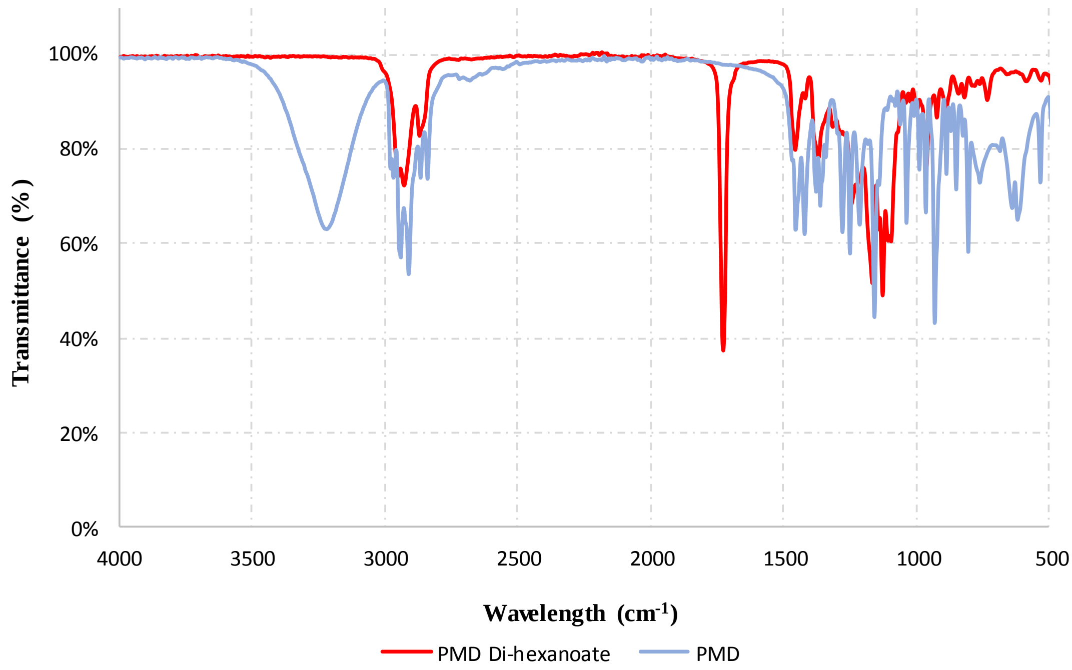

FTIR spectrum

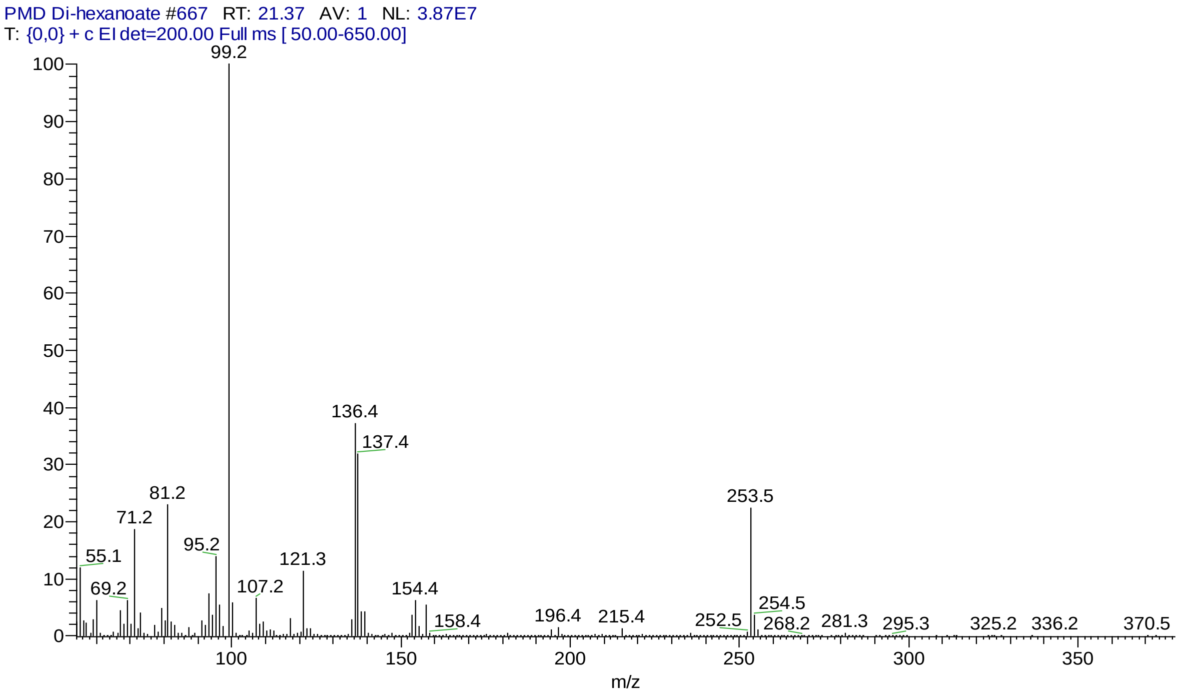

GC–MS spectrum

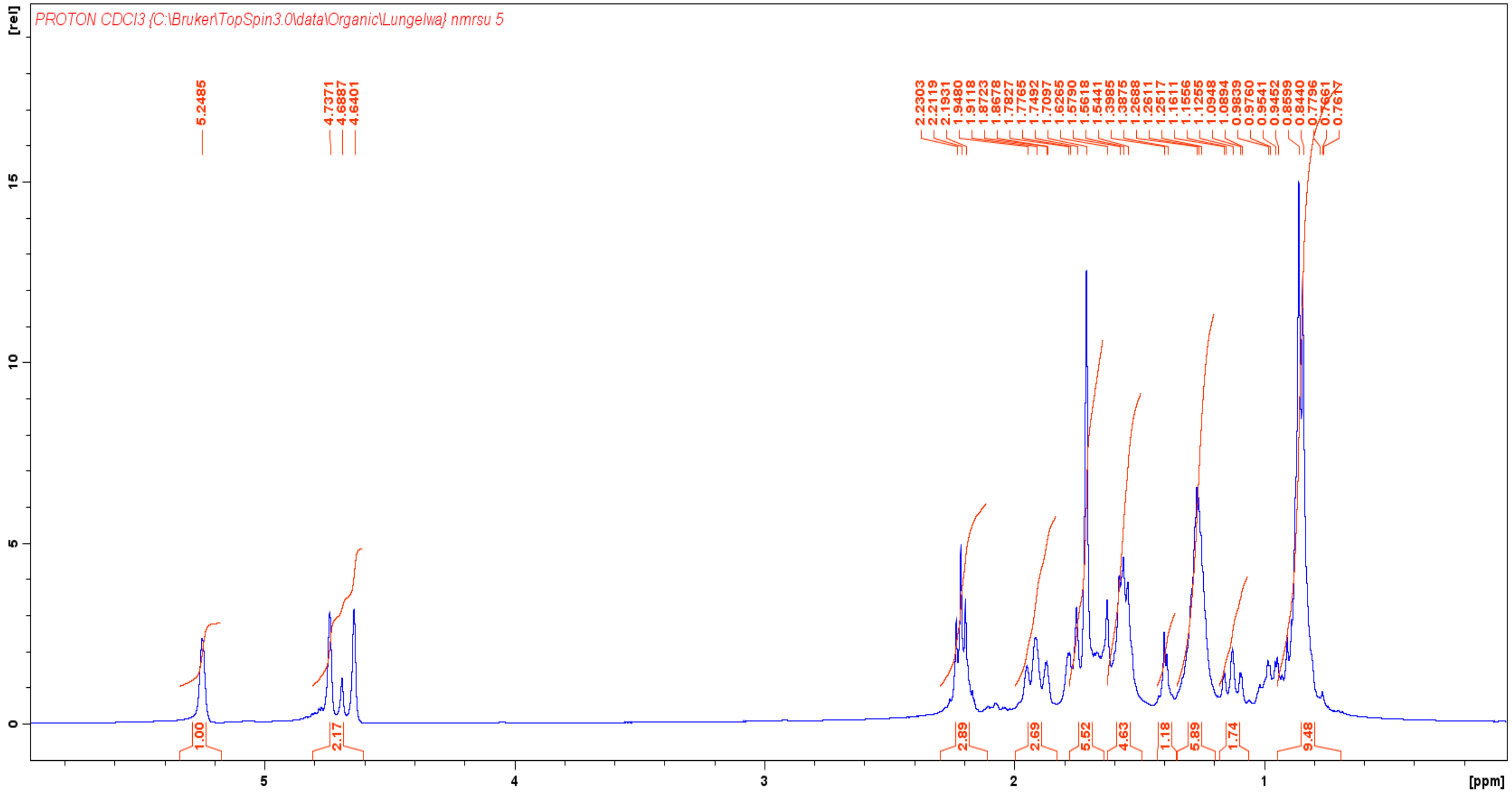

^{1}H NMR spectrum

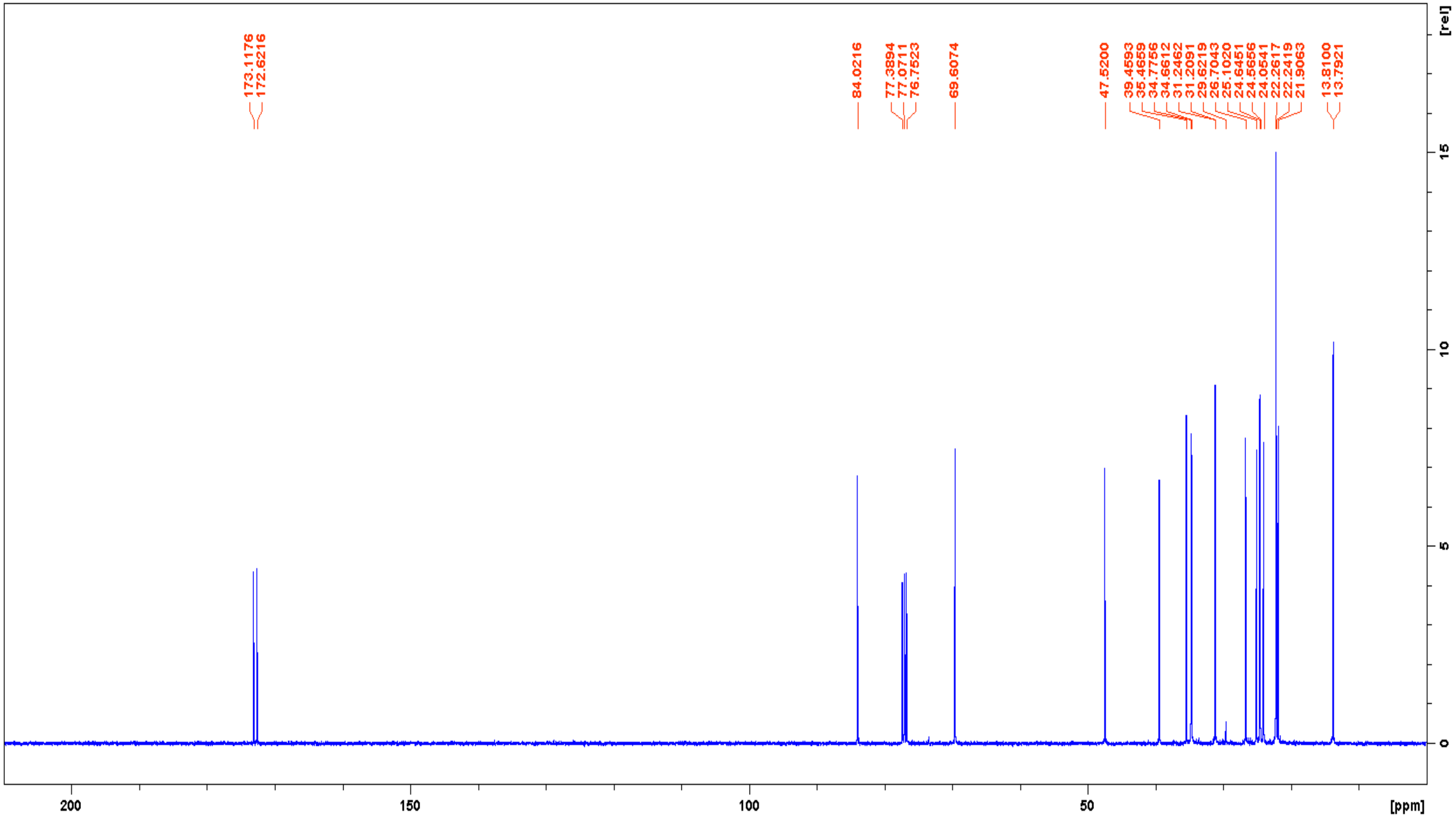

^{13}C NMR spectrum
